# 5G support for Industrial IoT Applications— Challenges, Solutions, and Research gaps

**DOI:** 10.3390/s20030828

**Published:** 2020-02-04

**Authors:** Pal Varga, Jozsef Peto, Attila Franko, David Balla, David Haja, Ferenc Janky, Gabor Soos, Daniel Ficzere, Markosz Maliosz, Laszlo Toka

**Affiliations:** Department of Telecommunications and Media Informatics; Budapest University of Technology and Economics, Magyar Tudosok krt., 1117 Budapest, Hungary; peto@tmit.bme.hu (J.P.); franko@tmit.bme.hu (A.F.); balla@tmit.bme.hu (D.B.); haja@tmit.bme.hu (D.H.); fecjanky@gmail.com (F.J.); soos2g@gmail.com (G.S.); ficee12@gmail.com (D.F.); maliosz@tmit.bme.hu (M.M.); toka@tmit.bme.hu (L.T.)

**Keywords:** 5G, IoT, Industry 4.0, ultra-reliable low latency communications, mobile edge cloud, virtualization, artificial intelligence, blockchain, private campus network, survey

## Abstract

Industrial IoT has special communication requirements, including high reliability, low latency, flexibility, and security. These are instinctively provided by the 5G mobile technology, making it a successful candidate for supporting Industrial IoT (IIoT) scenarios. The aim of this paper is to identify current research challenges and solutions in relation to 5G-enabled Industrial IoT, based on the initial requirements and promises of both domains. The methodology of the paper follows the steps of surveying state-of-the art, comparing results to identify further challenges, and drawing conclusions as lessons learned for each research domain. These areas include IIoT applications and their requirements; mobile edge cloud; back-end performance tuning; network function virtualization; and security, blockchains for IIoT, Artificial Intelligence support for 5G, and private campus networks. Beside surveying the current challenges and solutions, the paper aims to provide meaningful comparisons for each of these areas (in relation to 5G-enabled IIoT) to draw conclusions on current research gaps.

## 1. Introduction

The fifth generation of cell-based mobile communication architecture is commonly called 5G. It has been motivated by various factors, some are purely related to communications, such as serving highly populated areas with high-speed mobile access, and some are less related to communications, such as battery lifetime for over 10 years, among others. Traffic-related motivations include the expanding requirements for enhanced mobile broadband (eMBB), ultra-reliable and low latency, so-called critical communication scenarios (URLLC), and the envisioned massive machine type communication (MMTC), or mIoT, massive IoT (Internet of Things), traffic demands.

One of the new areas where cellular mobile communications enter due to 5G is industrial IoT, especially regarding ultra-reliable and low-latency communication needs. This paper aims to provide a comprehensive view on how 5G and related emerging methods and technologies intend to support the needs of industrial actors.

Note that this article focuses on the underlying solutions within and beyond the core network, while the specific challenges of the radio access network is outside of the scope for the current paper.

The contributions of this paper are the following.

- It compiles a state-of-the-art survey on 5G and related technologies that respond to the needs of industrial stakeholders—including those motivated to run private campus networks.
- It describes challenges and research gaps to be solved based on application-specific requirements.
- It provides an overview of some of the challenges already addressed or have solution examples.
- It adds emerging areas into the perspective, such as the tactile internet, AI (artificial intelligence) solutions, distributed ledger technologies, proposing that having such technologies on board can further support meeting industrial IoT targets when combined with the availability of 5G capabilities.

The current paper goes beyond the latest comprehensive survey so far [1] in terms of covering IIoT rather than IoT, and going into the details related to the involvement of emerging technologies: from production-related issues through mobile edge computing the edge-cloud, back-end virtualization, as well as security and smart service contracts to artificial intelligence and private campus networks. Some of these, such as the back-end, its virtualization, or smart service contracts, are rarely surveyed within the IIoT domain, although the underlying technologies and the results have great impact on IIoT applications. Nevertheless, the authors of [1] provided a high-level overview of the generic IoT-related 5G challenges, such as the architecture itself, scalability, massive deployments, interoperability and dense HetNets, security and privacy concerns, SDN and NFV issues, device-to-device communication, and energy efficiency; and listed related research trends at the time, so from such a perspective that survey is highly recommended for reading, as well.

## 2. IoT Requirements—Critical and Mass

**Figure 1:**
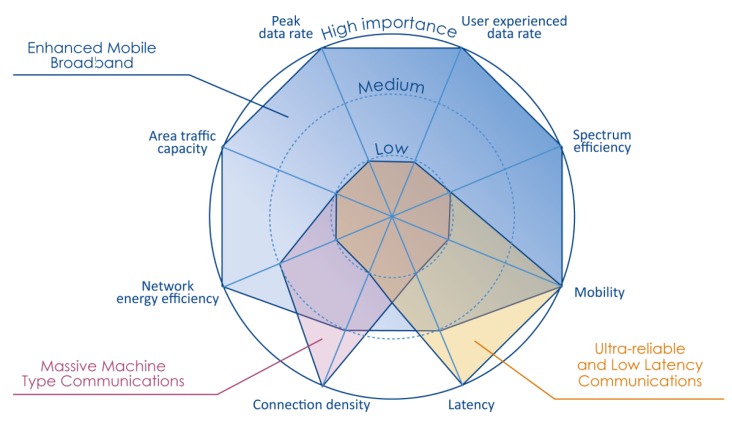
The importance of key capabilities in different usage scenarios [2].

The high level expectations of market actors on 5G are not as detailed as the 3GPP recommendation. Although the high level requirements were gathered from various sources, one of the earliest standards on that was ITU-R M.2083-0 [2]. When describing the importance of key capabilities in different usage scenarios, this standard was the first to define those three types of key communication scenarios: eMBB, URLLC, and MMTC. Their characteristics are summarized by Figure 1, which already shows the major requirements for mass IoT or MMTC traffic as (i) network energy efficiency and (ii) connection density; whereas, for URLLC traffic, mobility and latency.

Although the mass IoT or MMTC do not have high throughput expectation (when compared to eMBB), there are connection density requirements appear as high as 1 million endpoints per km^2^. This is not far from broadband access requirements in a crowd (half million users per km^2^), as Table 1 shows whilr providing examples of eMBB scenarios.

When discussing the requirements of industrial IoT applications, the requirements for real-time (low-latency) operation, high security, system interoperability and integrability, effective deployment and engineering, and proactive maintenance emerge [4].

The recommendation 3GPP TS 22.104 [5] details the requirements for cyberphysical control applications in vertical domains further. The level of detail for this quasi-standard is convincing: the standardized values for latency (0.5 ms to 500ms at different scenarios), high reliability (starting from 99.999% up to 99.999999%), and long-term reliability (with mean time between failures ranging from 1 day to 10 years) must ensure the wide applicability of 5G. This 3GPP TS 22.104 recommendation covers various performance requirements for both periodic and aperiodic deterministic communication (as well as nondeterministic communication), mixed traffic, clock synchronization, positioning performance, and network operation. The requirements are also elaborated on a per-scenario basis, covering various use-cases within factories of the future, electric power distribution, central power generation, as well as connected hospitals, and medical facilities (indeed, including clear requirements for robotic surgery, among others).

While Vehicle to Everything (V2X) communication is also expected to be the “killer application for 5G”—and the respective requirements are detailed in 3GPP TS 22.186 [6]—this area falls out of the focus of this paper, targeting Industrial IoT. Similarly, although rail communications have their own strict requirements elaborated in 3GPP TS 22.289 [7], this paper does not focus on the specific issues of this transport domain.

The rest of the paper is organized as follows. Section 3 provides a brief overview of 5G and some of its core technologies. Section 4 discusses 5G support for IIoT applications, especially those that utilize robotics. Section 5 introduces the current challenges and solutions in the Mobile Edge Cloud. Section 6 deep dives into the next architectural element and data center back-ends, whereas Section 7 surveys the general issues in virtualization network functions (VNFs). As a mandatory element of IIoT, security considerations are discussed in Section 8 The latest conceptual interworking in the field are blockchains with IIoT and the utilization of Artificial Intelligence in 5G—these are addressed in Section 9 and Section 10 respectively. The private campus network is a sensible manifestation concept for 5G in future factories; motivations and challenges are discussed in Section 11; whereas, Section 12 concludes the paper.

## 3. A Brief Overview of 5G

### 3.1. Requirements

The requirements and the current technological capabilities delineate the sensibly reachable targets for 5G. The recent recommendation on the “Service requirements for the 5G system” is described in TS 22.261 [3] by 3GPP. It formulates 32 different basic capabilities, defines the performance requirements, as well as security and charging aspects.

Clearly not all the listed service requirements are related to IoT, i.e., charging aspects are vaguely defined; and those are anyway outside of the scope of this paper, although the performance and security related ones are all relevant; as well as some basic capabilities. Those capabilities that are majorly relevant for IoT are the following.

- Network slicing,
- resource efficiency (management for IoT, bulk operations for IoT),
- efficient user plane,
- priority, QoS and policy control,
- network capability exposure,
- energy efficiency,
- QoS monitoring (especially for URLLC, vertical automation communication, and eV2X services),
- non-public networks,
- positioning services, and
- cyberphysical control applications in vertical domains.

The performance-related requirements are addressed from various aspects throughout the article. According to TS 22.261 [3], these include high traffic density, low latency, high reliability, high accuracy positioning, high availability IoT traffic, and Key Performance Indicators (KPIs) for the User Equipment (UE) to network relaying in 5G system. The actual target measures and KPIs are detailed in the various tables of TS 22.261 [3].

High reliability is especially important for 5G, since it is a key distinguishing feature when compared to architectures that use non-licensed radio spectrum, or legacy, evolutionary-engineered heterogeneous networks.

A vast body of research on 5G high reliability tackles every aspect of the well-known network performance problems: reliability, latency, and bandwidth. Providing high reliability for novel 5G services is actually even greater at the advent of a geographically distributed system. The difficulty stems from the fact that wide-area networking and all its well-known issues come into play and affect the performance of the applications. When addressing these issues, the authors of [8,9] considered the replica and virtual function placement, to achieve lower migration time. As a further step, authors of [10] investigated the fog resource provisioning problem for deadline-driven IoT services to minimize the cost considering the probability of resource failures. They considered that VM failures are temporary and recoverable.

### 3.2. 5G Network Architecture in Brief

**Figure 2:**
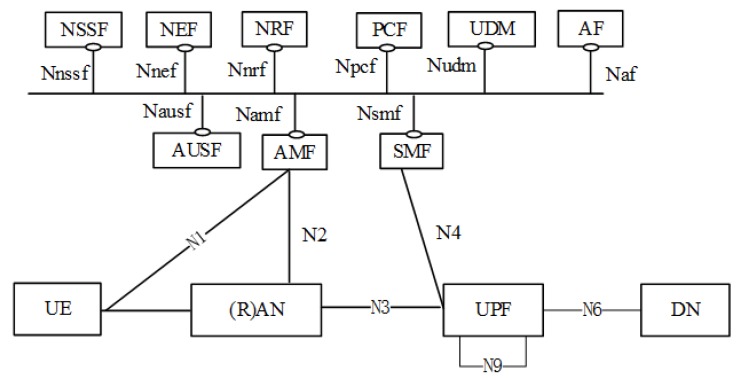
The reference architecture for 5G—non-roaming case [11].

The generic architecture for 5G is depicted by Figure 2. The main functional entities are the following.

- User Equipment (UE)—is the actual end-device;
- (Radio) Access Network ((R)AN)—can include various technologies; the next-generation, 5G-specific radio controller and transceiver is the gNB;
- User Plane Function (UPF)—provides packet routing, forwarding, and inspection; it is the anchor point for mobility management, and it is the user plane part of policy rule enforcement—among other roles;
- Data Network (DN)—e.g., operator services, Internet access or 3rd party services;
- Authentication Server Function (AUSF)—supports authentication for 3GPP access and untrusted non-3GPP access;
- Access and Mobility Management Function (AMF)—is the termination point for non-access stratum ciphering and integrity protection, provides access authorization and authentication functions, manages mobility, registration, connections, reachability, and many more;
- Session Management Function (SMF) is responsible for various session-related control functions, including session establishment, modification, and release, including tunnel maintenance, among others;
- Network Slice Selection Function (NSSF)—selects the slice instances to serve the UE, and determines the related NSSAI (Network Slice Selection Assistance Information);
- Network Exposure Function (NEF)—provides exposure of capabilities and events, secure provisioning of information from external applications, translation of internal-external communication, and many more;
- Network Repository Function (NRF)—supports the service discovery function and maintains the Network Function profile;
- Policy Control Function (PCF)—supports unified policy framework to govern network behavior, provides policy rules and accessess policy information at UDR for relevant policy decisions;
- Unified Data Management (UDM)—is responsible for managing user data for various services, including access authorization, generation of authentication credentials, user identification handling, subscription management, SMS management, among others;
- Application Function (AF)—interacts with the core network in order to provide services such as application influence on traffic routing, or interactions with the policy framework for policy control.

### 3.3. Network Slicing

**Figure 3:**
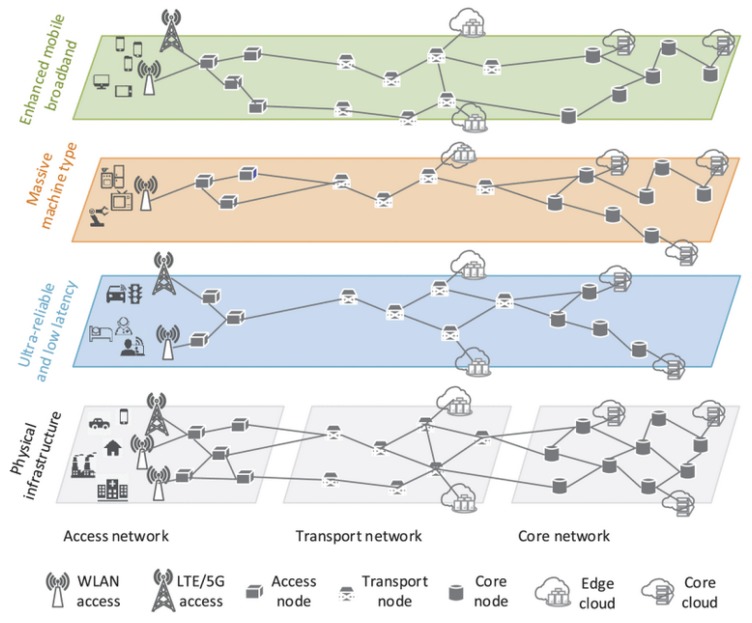
An architectural view of the 5G network slicing [13].

Network slicing has become a key concept in 5G enabled systems. With slicing, the service providers are able to support novel businesses services with diverse requirements in an efficient way. What does network slicing really mean in 5G? It can be perceived as one network sliced into multiple instances, where each one is optimized for a specific requirement and/or specific application/service [12]. Figure 3 provides a generic architectural view on this. Slicing enables deploying several virtual networks on a physical infrastructure, allowing resource isolation and customized network operations. For multi-tenant systems, these two features are obligatory. From the infrastructure point of view, a set of dedicated or shared resources, either of physical or virtual, are allocated with network slicing to the appropriate tenants by introducing a network hypervisor.

As novel 5G services have diverse requirements, network slices need to combine a set of network and cloud resources, such as bandwidth, network functions for processing and storage, among others, to fulfill these conditions.

### 3.4. SDN, NFV and Resource Orchestration Approaches in 5G

The trend of virtualization leads to the appearance of cloud-native applications and infrastructure managers that ensure the seamless operation of those applications. Today’s most widely used resource and service managers, e.g., Kubernetes [14] that manages containers primarily for web applications, provide automatic scheduling, scaling, and self-healing features. Most of these solutions, however, target solely data centers, and are not designed for a geographically distributed infrastructure. Moreover, they often fail to employ such scheduling policy that guarantees the low latency the novel applications demand.

A dedicated component, namely, the resource orchestrator (RO), is in charge of managing the underlying resources and finding the proper placement of software components realizing the service. Following ETSI’s terminologies on Network Function Virtualization (NFV) [15], the software modules composing the network service are referred to as Virtual Network Functions (VNFs).

VNF (and NFV) is often mentioned together with Software Defined Networking (SDN), especially when discussing 5G core network resource optimization and slicing [16]. On one side, SDN is originated from the telecom operators’ domain with the intent of separating the rigid and interdependent structure of control and forwarding planes in networking equipment. On the other hand, VNF/NFV has emerged from the data center and resource optimization community aiming to provide flexibility in the network functions. These communities nowadays work together in solving challenges brought by the requirements of 5G slicing [17]. The main concepts and respective challenges have recently been summarized in [16].

A key element of this infrastructure is the resource orchestrator, which can be considered as a component encompassing orchestration related tasks, and in ETSI’s architecture it appears both in the Virtual Infrastructure Manager (VIM) and in the NFV Orchestrator (NFVO). In general, RO assigns VNFs composing the service to compute resources and also allocates paths between connected VNFs.

A novel RO (or a hierarchy of ROs) that can efficiently manage underlying resources in cloud-edge/mobile edge/distributed cloud computing environments is an inevitable future component with challenging tasks. It must be able to jointly handle compute and network resources in a tightly integrated framework and it must be aware of network characteristics besides compute capabilities. Furthermore, the requested network services have to be created on-the-fly within seconds. Two different design approaches can be applied to achieve such features. On the one hand, on top of VIMs and network controllers, a higher level orchestrator, i.e., the NFVO, can be added which is able to integrate different resource domains. This solution results in a hierarchy of ROs and the cooperation of VIMs and NFVO yielding larger deployment time and the need for strictly defined external APIs. Multi-provider scenarios require this approach. On the other hand, the VIM itself can be extended with network awareness and with the detailed view on network resources. With such an upgrade, the additional NFVO becomes unnecessary for single-provider setups where resources belong to the same operator and by these means, the orchestration and deployment time can be reduced significantly [18].

## 4. 5G Support for Industrial IoT Applications Utilizing Robotics

5G provides capabilities that modern industrial applications need. Robotics has a special importance due to its URLLC requirements. In this section, we present and compare several recent papers that show industrial use cases for 5G technologies to identify the respective challenges.

### 4.1. Industry 4.0

The key capabilities of 5G (supporting eMBB, URLLC, and MMTC) act as an enabler for Industry 4.0. According to [19], Industry 4.0 is defined as the current trend of automation and data exchange in manufacturing technologies. Its main research domains include cyberphysical systems, the Internet of Things, cloud computing and cognitive computing. Industry 4.0 creates what has been called a “smart factory”. Within the modular structured smart factories, cyberphysical systems monitor physical processes, create a virtual copy of the physical world and make decentralized decisions. The current 4G/3G limitations can be described as reliability of wireless connections, energy consumption of devices, end to end delay, supporting extreme density of devices. 5G removes these limitations, enabling use-cases that were not possible before. These can be grouped into the following categories: Time Critical and Reliable Processes (e.g., real-time monitoring, vision controlled robot), Non-Time Critical Communication (logistic process involving assets and goods, quality control process or data acquisition), and Remote Control of Factory (remote workers, augmented reality).

The authors of [20] discuss the application of 5G communication technology in an industrial environment as 5G offers characteristics essential for industrial use-cases such as robustness, ultralow latency, high data rates, and massive number of devices. The paper introduces an Industrial Demonstrator Platform consisting of a mobile robot and ten production modules. In this work it is shown that 5G can be applied in a real-world application industrial environment with heterogeneous data sources. The capabilities of 5G communications show great potential for certain industrial applications as an alternative for wire-based transmission.

When broadening the view for 5G for industrial applications, the authors of [21] first identified more advantages of 5G than the main three (eMMB, URLLC, and mMTC): Mobility support, Energy efficiency, Security, Economy of scale, and core networking changes: Softwarization and virtualization, SDN, and Mesh networks. The paper further lists several use cases and technical requirements for the main three capabilities: Infrastructure retrofit, Mobile robots, Inbound logistics for manufacturing, flexible and modular assembly area, plug-and-produce, Massive wireless sensor network, and process monitoring. After this, the paper then proposes a communication network architecture that can fulfill the requirements of the listed use cases, and integrates it into reference architectures for Industry 4.0 (Reference Architectural Model Industrie 4.0) and IIoT (Industrial Internet Reference Architecture).

The authors of [22] demonstrate the application of 5G communication technology in robotics, how the URLLC capabilities of the 5G can facilitate a distributed robotics control system. The proposed approach allows offloading of time-critical, computational exhaustive operations onto a distributed node architecture such as a cloud server using 5G URLLC communication between the cloud and the robot. This approach is demonstrated using a real-world application performed by a mobile robot.

**Figure 4:**
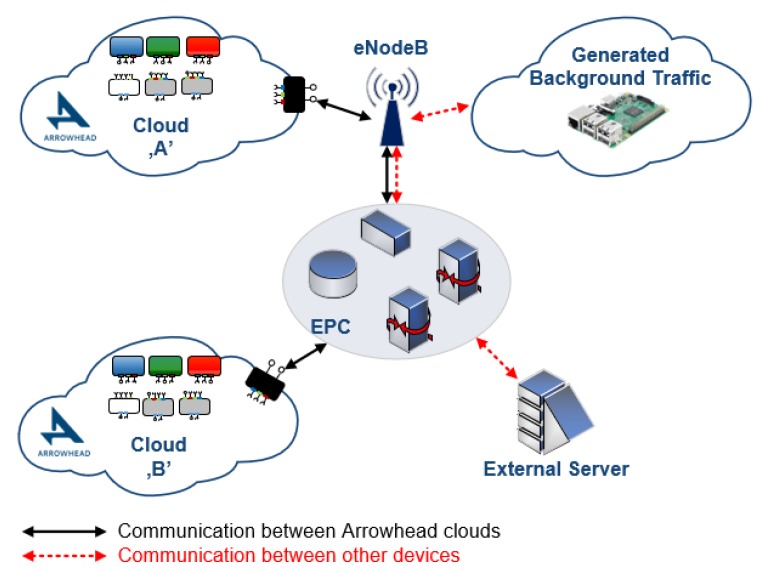
Benchmarking infrastructure for measuring heterogeneous local automation cloud communications capabilities [26].

Digital production, product lifecycle management, and supply chain management are the three pillars of the collaborative European project, Productive4.0 [4]. The Arrowhead Framework [23,24] is used throughout various use-cases here, in order to provide interoperability, integrability and ergonomic engineering capabilities. The real-life measurements of Arrowhead-based local automation clouds [25] show that although NB-IoT outperforms Cat-3 or Cat-M1 infrastructures under harsh conditions [26], LTE-based technologies cannot satisfy industrial requirements. Even QCI-prioritization cannot help the LTE-enabled end-devices to communicate with less than 10ms delay (not to mention jitter) [27]. On the other hand some recent, practical 5G measurements [28] confirmed that devices are able to communicate with less than 5ms end-to-end delay under basic (not tuned) configurations, even if background traffic utilized 50% of the network capabilities. The benchmark architecture of these measurements is shown by Figure 4.

### 4.2. Cyberphysical Systems and Industrial IoT

When discussing data collection, analysis, and feedback from various industrial endpoints, and less about their enterprise effects, Cyberphysical Systems supporting the Industrial IoT (IIoT) come into focus.

Cyberphysical-based manufacturing systems (CPMS), or in other terms, cyberphysical production systems (CPPS), which are designed to realize smart management in manufacturing physical space through data modeling, integration, fusion, simulation, analysis, and decision-making are also impacted by 5G. Based on the respective advantages of 5G application scenarios and the basic architecture of CPMS, [29] proposes a 5G-based IIoT architecture. Furthermore, the paper describes implementation methods of manufacturing scenarios and technologies using eMMB, URLLC, and mMTC, and it analyzes the key technologies and challenges of 5G-based IIoT, as well.

Obviously IIoT is not just for manufacturing; the potential usage of IoT technology for various construction project processes is presented in [30]. This article proposes a construction domain framework, which is used to identify and discuss the barriers raised by connectivity issues, which are then overcome by the influence of 5G technologies. Using an example, the paper presents a comparative quantitative analysis of sample processes to show the potential advantage that 5G technology will bring over 4G.

IIoT can also be used to implement positioning using 5G, as shown by the authors of [31], among others. The paper discusses several features of 5G positioning in the context of IIoT applications, which demand high accuracy of position information. It summarizes the main opportunities to come with 5G networks, such as huge available spectrum, small cell networks, Multiple Input Multiple Output (MIMO) antennas and beamforming, and points out the challenges in the context of robot 5G positioning. It presents a case study for the localization of an indoor robot in a multi-wall multi-floor scenario, based on various carrier frequencies and access node densities. The paper finds that sub-meter positioning accuracy required for most of the future industrial applications is theoretically achievable via a combination of small cell networks, mm Wave carriers and antenna arrays, but practical issues such as node synchronization, connectivity and ultra dense network deployment costs have to be tackled.

### 4.3. Tactile Internet

The core concept of tactile internet is that it allows performing operations and manipulation of physical objects over distance, including fine-grained physical interactions. Recent research works [32,33] discuss novel applications with which an underlying technology, such as tactile internet, will change health care, education, manufacturing, smart grids, etc. Tactile internet will let humans control real and virtual objects in a wireless manner. The key challenge in tactile internet realization is that it has to adapt to human reaction times. Reaction times of approximately 100, 10, and 1 ms is required for auditory, visual, and manual interaction, respectively. One can easily see that the crucial requirements for tactile internet are ultra-responsive and ultra-reliable network connectivity for achieving acceptable user experience.

An overview of the 5G communication features and the 5G capabilities to address tactile internet services and haptic interactions and communication is provided within [34]. These include a 5G system architecture that builds on a flexible software-based network design based on a distributed cloud infrastructure; also, a 5G wireless communication design toward reliable and low latency communication. Finally the paper describes the first trials and experiments the authors made with 5G-enabled tactile internet services.

An interesting use-case of tactile internet is telesurgery; the paper [35] introduces a telesurgery robot based on the 5G tactile Internet and artificial intelligence technology. It explains the architecture, composition, characteristics, and advantages of telesurgery. Then, it presents optimization schemes based of 5G technology: edge-cloud integration (Section 5), network slice (Section 3.3), and AI in edge-cloud. Finally, the paper discusses the open issues of the presented telesurgery system regarding the ultra-high reliability, AI-enabled surgery robot, communication, and security.

### 4.4. Miscellaneous Usage of 5G Technologies for Industrial Purposes

**Network Function Virtualization (NFV)**—described in Section 3.4—can be used to support smart manufacturing in various ways. In papers [36,37] the authors present a detailed use case that reflects the needs of real-world manufacturers using the experience from a large-scale manufacturing company. They propose an architecture with specific network services and virtual network functions that realize the use case in practice. Their experimental results indicate that a fully virtualized smart manufacturing use case is not only feasible, but also reduces machine interconnection and configuration time and thus improves productivity by orders of magnitude. Then, they show a real-world smart manufacturing application entirely implemented using NFV concepts, and a lightweight prototyping framework that simplifies the realization of vertical NFV proof-of-concepts, they demonstrate how NFV services can simplify machine data collection, aggregation, and analysis.

**Network slicing**, introduced in Section 3.3, can be of significant help for industrial applications. The authors of [38] present a platformized the 5G system aiming at developing reconfigurable Industrial Internet environments for smart factories in order to support dynamic production processes, while optimizing incurred cost and enabling a high level of remote monitoring and control. It proposes a framework based on network slicing for Industry 4.0. The proposed framework is validated in three different realistic use cases, which clearly point out the advantages of the envisioned solution: Remote Industrial Production Monitoring, Remote Equipment Maintenance, and Dynamic Industrial Manufacturing.

Network slicing can also be used in autonomous driving. As detailed in [39], SDN, NFV, and network slicing can enhance the QoS of autonomous driving applications. It presents a theoretical analysis of the propagation delay and the handling latency based on GI/M/1 queuing system. Simulation results show that the framework meets the low-latency requirement of the autonomous driving application as it incurs low propagation delay and handling latency for autonomous driving traffic compared to best-effort traffic.

**Multi-access Edge Computing (MEC)**, as described in Section 5, formerly known as Mobile Edge Cloud, allows virtualization of services in factory production achieving higher service reliability and smaller cost of industrial equipment. The MEC approach allows efficient resource planning through a dynamic extension or reduction of allocated resources [40]; and this approach can improve the reliability through dynamic management of the allocated computational resources between different cloud services. The paper shows the concept of MEC for two industrial use cases. The use case “remote control of the robot arm” can be acquired to perform the work in dangerous environments. The use case “cooperation of two robot arms” extrapolates to the cooperation between multiple robot arms on mutual work on the production line. The use-cases were realized in a real-world demonstration.

### 4.5. Lessons Learned

In Table 2, we summarize the uses cases described in papers presented in the current Section 4. The table shows how many use cases were discussed for the main 3 features of 5G (eMBB, URLLC, mMTC) and what other 5G technology use cases were described. In the realization column, we display how the given paper realized the use cases presented. The papers present several use cases in different environments (manufacturing, telesurgery, positioning, and construction management) for many 5G technologies showing that 5G can be used widely in the industry.

### 4.6. Identified Research Gaps

The discussed articles present many diverse use cases, and suggest architectures that implement them, but not many of them show real world demonstrations. It would be great to see more use case realizations showing industrial users that 5G can fulfill its promises for Industry 4.0.

Other important aspect that is not considered in depth is security, because for many use cases, especially in manufacturing, security is of paramount importance.

## 5. 5G and the Edge Cloud

**Figure 5:**
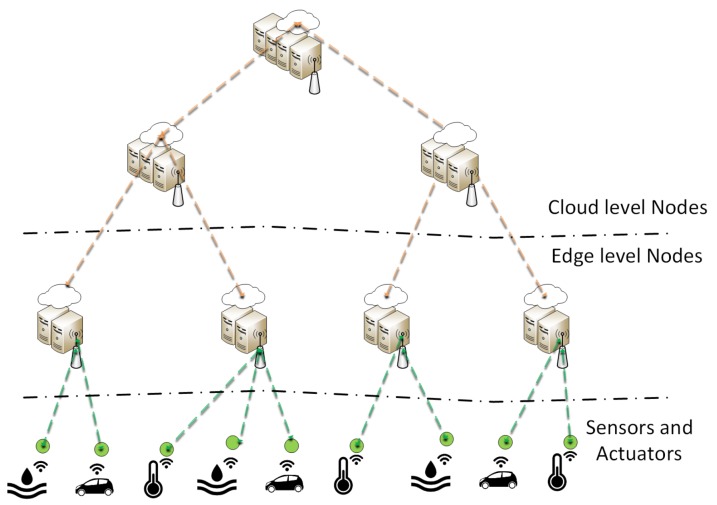
The OpenFog reference architecture: N-tier fog deployment [42].

Cloud-edge (or fog) computing [41,42] and MEC [43] are novel concepts extending traditional cloud computing by deploying compute resources closer to customers and end devices. Figure 5 shows a high-level view of the OpenFog reference architecture. This approach, closely integrated with carrier-networks, enables several future 5G applications and network services, such as novel Industry 4.0 use-cases, Tactile Internet, smart cities, or remote driving [44]. Edge resources provide execution environments close to users in terms of latency (e.g., in mobile base stations). By these means, on the one hand, customers’ devices can offload computational tasks to this edge environment instead of consuming their local resources. On the other hand, latency critical functions can also be offloaded from central clouds to the edge, enabling critical machine type communication which is required by various envisioned services.

Several recent articles [12,43,45,46] present the use case possibilities, the fundamental key enabling technologies and various orchestration options of edge computing. OpenFog consortium [47] and ETSI [48] have in-progress standardization activities, in which both organizations develop their respective MEC reference architectures.

In this section, we present those 5G use-cases, that benefits the most from the edge computing concept and summarize the related research works. At the end of Section 5 we provide a comparison of the presented applications and the requirements they pose against 5G network.

### 5.1. Data/Video Analysis in the Edge

In [49], the authors stated that large-scale video analytics could be the “killer application” of edge-clouds. The authors proposed a video analytics system called Rocket, which can operate real-time and has low resource cost. Although their system is designed for generic purposes, the initial application focuses on traffic planning. The rocket tries to estimate the video queries’ resource-accuracy profile and optimize the video pipeline. The resource manager provides the access to all resources (CPU, GPU, network, etc.) through geodistributed executors to the query pipelines. To further reduce their system’s resource demand, the authors developed additional application-level techniques such as intelligent frame selection and intelligent feed selection.

Authors of [50] provide a highly dynamic system, that is, a modular based on a scalable architecture that relies on lightweight virtualization techniques. The proposed architecture consists of three main layers: sensing (edge devices), mediation (gateways), and enterprise layer (cloud). This architecture is validated through a typical smart home scenario, where different IoT end-devices communicate with the cloud through a gateway (GW), acting as a mediator, caching the data locally and lightening the edge-devices’ resource usage.

Firework [51] provides distributed data processing and sharing via a virtual shared data view and service composition for Internet of Everything (IoE) applications. Firework collects and concatenates data and predefined functions from multiple stakeholders and data owners to a virtual shared data set with the care of data privacy protection. This novel framework can split an application into “subservices” enabling users to subscribe for intermediate data and create new applications by leveraging existing “subservices”. In addition, it provides an easy-to-use programming interface for both service providers and end users. The experimental results of [51] article demonstrate how Firework can reduce response latency and network bandwidth cost for a video analytics application compared to a cloud-centric solution.

### 5.2. eXtended Reality (XR) and Industry 4.0 Applications

Extended reality (XR) is one of the most emerging use cases nowadays. Virtual reality (VR) replicates a real-world environment or creates an imaginary world to involve users in an entirely virtual world. Augmented reality (AR) techniques fuse digital content with physical environment in real-time. Various fields can use these technologies, such as health care, entertainment, education, industry, etc. The future production systems, envisioned by Industry 4.0, has stringent requirements. They must be flexible, scalable, and easily reconfigurable systems supporting product-independency, universal manufacturing processes and capacity scaling, depending on the actual demand, is also a key enabler feature.

The authors of [52] present Navantia’s (one of the 10 largest shipbuilder company in the world) industrial AR (IAR) architecture, which relies on cloudlets [53] and on the fog computing paradigm. Note that edge computing is really close to fog computing and, in fact, some authors consider fog computing as one of the possible implementations of edge computing. In the article the proposed architecture is validated with real-world Microsoft HoloLens applications. The authors show that fog gateways are the fastest when transferring small payloads in terms of response delay, but a cloudlet is faster for medium and large IAR payloads.

In [54], the components of general AR applications, the fashionable AR devices, and several existing techniques for overcoming the thorny latency and energy consumption problems are reviewed. The authors propose a three-layered hierarchical architecture, based on the MEC reference architecture. During the simulations, they considered a scenario, where multiple mobile devices, near the same base station, execute AR applications simultaneously. The simulation results show how they can significantly improve the latency and energy performance when compared against existing baseline schemes.

The user equipment has limited resource capability, therefore it is difficult to meet the stringent service requirements, such as latency and reliability. One solution can be a scenario, when some part of the AR task is offloaded to the edge of the network. In [55], a scenario is considered, where multiple edge nodes cooperate to complete the AR tasks. The dependency between the components of each task is modeled with a directed acyclic graph through code partitioning. The objective of the proposed algorithm is to minimize the service failure probability (SFP) of the MEC-enabled AR service with the consideration of reliability and latency. The proposed heuristic algorithm in the article can significantly improve the probability to fulfill the targeted SFP in various network conditions.

In [56], an intelligent resource scheduling strategy is proposed with an underlying hybrid computing framework. The computing system consists four layers for supporting artificial intelligent tasks: device computing layer, edge computing layer, cloud computing layer, and SDN layer. Then a two-phase algorithm is demonstrated for scheduling edge layer resources with the consideration of latency requirements. For the evaluation, the authors considered the following experiment; a mobile robot with an industrial camera moved along with a fixed trajectory to monitor the operation of equipment of the constructed Industry 4.0 platform. This robot periodically captured pictures and then calculated the images boundary for the next work process. Then, the running state of the equipment is judged by a neural network. Finally, the authors concluded that their resources scheduling technique can accelerate the implementation of Industry 4.0 and smart factory.

### 5.3. Caching in the Edge

Many previous works on mobile edge computing focus on how computational tasks can be offloaded to resources in the network. In contrast, the authors of [57] introduce a novel concept of task caching, which refers to caching the completed tasks and their related data in edge cloud. The authors investigate and formulate a joint optimization problem related to task caching and offloading in edge cloud with the consideration of computing and storage resource constraints. An efficient algorithm, called task caching and offloading (TCO) is proposed for resolving the problem. With simulation experiments the authors present the performance of their algorithm, which shows that it outperforms other solutions in terms of less energy cost.

In [58], Edge-CoCaCo is presented, a joint optimization of computation, caching, and communication on the edge cloud. The authors propose a solution to that optimization problem. The Edge-CoCaCo model deals with two problems: computational task cache placement problem and task offloading problem. Although the latter problem is well known, the former one refers to a decision problem of whether to cache the computational task on the edge or not. The simulation results show that Edge-CoCaCo can decrease the delay of computation-intensive and rich-media tasks in an edge cloud environment.

### 5.4. Smart Vehicles

Vehicles are used in industry for logistical reasons, within warehouses and in-between sites. Although the current article does not mean to be comprehensive in this area, some elementary issues are discussed in connection with the edge cloud.

In [59], electric vehicles cloud and edge (EVCE) computing is presented. EVCE is a network paradigm, which involves seamless connections in heterogeneous vehicular contexts to aggregate multiple geographically distributed electric vehicles (EVs) into a common resource pool. In EVCE computing, information and energy flow are dynamically exchanged during the vehicle-to-anything communications, including vehicle-to-grid (V2G), vehicle-to-infrastructure (V2I), and vehicle-to-vehicle (V2V). The purpose of this flow dynamical flow exchange is to achieve collaborative data sensing, information analyzing, and energy sharing. The article prioritize security issues for both information and energy interactions in EVCE computing. The authors define blockchain-inspired data coins and energy coins to achieve distributed consensus in context-aware vehicular applications. Finally, security schemes are proposed for cloud and edge computing to set up perspectives on vehicular applications.

In [60], an edge computing-enabled computation offloading method, named ECO, is proposed. Although the computing tasks in the Internet of connected Vehicles (IoV) paradigm can be offloaded to the edge computing devices (ECDs), the wireless communication for computation offloading increases the risk of privacy leakage, which may lead to virtual vehicle hijacking or tracking, etc. ECO solves this privacy conflict in computation offloading challenge with novel privacy preservation technique for IoV. A genetic algorithm is adopted to realize multi-objective optimization to reduce the execution time and energy consumption of ECDs and prevent privacy conflicts of the computing tasks.

Integrating fog computing and vehicular networks, vehicular fog computing (VFC) is promising to achieve real-time and location-aware network responses. The authors of [61] propose a VFC model with three layers. Their model enables distributed traffic management in order to minimize the response time of citywide events collected and reported by vehicles. Furthermore, the authors formalize a VFC-enabled offloading scheme as an optimization problem by leveraging moving and parked vehicles as fog nodes. Finally, the proposed model is validated with a taxi-trajectory-based performance analysis, coming from a real-world scenario.

In [62], the authors envision connected and autonomous vehicles (CAVs) as sophisticated computers on wheels. CAVs will have on-board sensors as data sources and there will be variety of services running on top to support autonomous driving or other functions. These envisioned novel services, especially the machine learning based applications, are expensive from the computational point of view. The authors propose an Open Vehicular Data Analytics Platform (OpenVDAP) for CAVs, which is a full-stack edge computing based platform including computing/communication unit in the CAVs. Furthermore, in OpenVDAP, an isolation-supported and security and privacy-preserved vehicle operation system is proposed. The authors created an edge-aware application library, which defines how to access and deploy edge-computing based vehicle applications. A novel scheduling strategy is presented, which enables CAVs to dynamically detect each service’s status and the optimal offloading destination so that each service could be finished within an acceptable latency and limited bandwidth consumption. OpenVDAP is an open-source platform that offers free APIs and real-world vehicle data to the researchers and developers in the community.

### 5.5. Lessons Learned

In Table 3, we summarize the requirements posed by the novel 5G services and use-cases presented in Section 5. These given values come from the related research efforts, where we selected the minimum delay and the maximum bandwidth requirements as reference values.

Most of the presented use-cases focus on decreasing the delay for their services; furthermore, several applications are aiming to reduce the bandwidth consumption in the wide area network (WAN). Although the use-cases have a wide range of requirements against the network capabilities, one can easily see that the delay requirements are strict in the sense that it has to be less than 100 milliseconds for every application.

Further feature capabilities are presented in our comparison: user mobility and energy consumption, which show whether any of the presented solutions take care of the users’ mobility capability, or tries to minimize the energy consumption of the devices. One can see that these novel 5G services requires very diverse features, which means edge computing supports wide range of new 5G applications.

### 5.6. Identified Research Gaps

As we see the research community have not identified the “killer application” of 5G and edge computing. Therefore many of the service requirements remain hidden beside the low latency. We believe the present solutions need to use advanced techniques, which they are not prepared yet, to fulfill these novel requirements. Furthermore, one of the key challenges comes from the fact that recent articles envision diverse topology scenarios, even for the same application or system. The realization and standardization of an edge cloud or 5G hierarchy will be beneficial for the research efforts.

## 6. Network Solutions at Data-Center Back-Ends to Meet Performance Requirements of 5G

The increased performance of 5G requires higher QoS guarantees from the back-end data-centers. This especially affects the networking part of the cloud data center, amplifying the need for applying different network acceleration technologies. These challenges may apply to the core, the edge cloud, and the cloud infrastructure of 5G, depending on the supported IoT applications.

### 6.1. Kernel Bypassing Network Solutions

**Figure 6:**
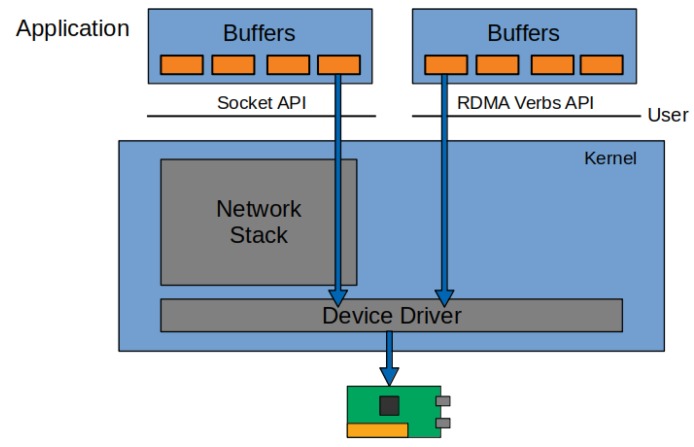
Kernel networking vs. Kernel bypassing.

The performance of the traditional kernel networking stack cannot meet the increasing requirements of 5G. Several papers had shown that kernel bypass networking, proposed by [63] among others (see Figure 6), significantly outperforms the traditional kernel based solutions. In [64], the authors showed that Remote Direct Memory Access (RDMA) and Intel’s Data Plane Development Kit (DPDK) shows a huge performance increase to the kernel network solutions. However, to reach the best performance, RDMA and DPDK should be used in poll mode, which of course leads to high CPU utilization. However, DPDK shows unstable operations after a few millions of packets when using it in event mode. On the other hand, RDMA requires special hardware; therefore, it might be inconvenient to implement a heterogeneous infrastructure.

### 6.2. Offloading Applications to the NIC

To mitigate the overwhelmed CPU usage of low latency network solutions, some functions had been offloaded to the network hardware. Initially, FPGAs had been utilized for these tasks; however, most of the developers are not familiar with hardware description languages (HDLs). Therefore, in [65], a solution is introduced that implements network functions by using a C-like language that are compiled into low-level HDLs for FPGAs.

On the other hand, in [66], the accelerated networking of Microsoft Azure is introduced, implemented on top of FPGA-based NICs, reaching a consistent <15 μs VM-to-VM TCP latency and 32 Gbps throughput. However, by using FPGAs only stateless utilities can be implemented; nevertheless, the need has increased for solutions supporting stateful applications onto the network hardware. For this reason, the use of smart NICs are getting more and more relevant. Smart NICs do not have enough compute resources to run large monolithic applications; however, according to the trend, virtualization technologies running in cloud environments are getting more and more granular. Therefore developers can implement highly distributed services of small components. Exploiting this Smart NICs, can cope with particular building blocks of a higher level service.

According to [67], by offloading microservices to smart NICs, the energy efficiency of the compute cluster could be improved by 3× and the cost efficiency up to 1.9×, by sacrificing only up to 4% latency for common microservices.

However, a recent research [68] revisited the field of smart NICs by comparing four commodity smart NICs by examining their offloading performance. The authors also implemented a framework—iPipe—for offloading distributed applications onto the network hardware, of which core implements a scheduler that is able to schedule tasks between host CPU cores and the smart NIC’s cores. By using iPipe, one can achieve considerable CPU and latency savings.

### 6.3. Using GPUs in Cloud Systems

There is an increasing need for using GPUs in the cloud as, e.g., deep learning and AI applications can gain performance using them. An initial and simple solution is to utilize PCI-passthrough capabilities of the host computer as it is shown in [69]; however, this solution does not let the GPU resources to be shared among several virtualized environments, on the other hand, the authors only showed the implementation and performance results coupled with the Xen hypervisor.

**Figure 7:**
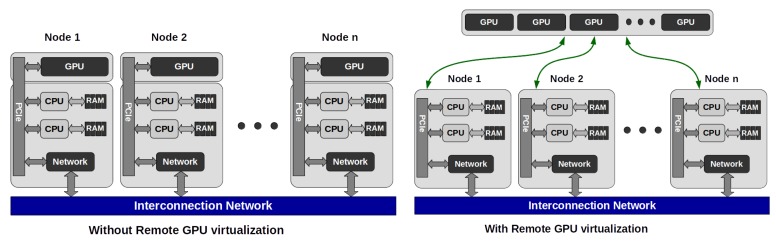
Local GPUs vs rCUDA architecture.

To solve this issue, the authors of [70] introduce a solution, rCUDA, in which case the video hardware can be shared among several virtualized environments by using a client-server based solution, making available utilizing the resources of remote GPUs supporting the use of hardware, located in compute nodes that are running virtualized environments leaving the GPU resources idle. On the other hand, more GPU resources can be assigned to an application than in the case of using only local GPUs. Such a system is also capable of rescheduling the video hardware resources, in case of a blocking CPU phase, to other remote applications leading to higher GPU utilization. The rCUDA architecture can be seen in Figure 7. According to the results, this can lead the reduction of energy consumption up to 40%. Furthermore, another great advantage of this solution is that no source code modification is required.

**Figure 8:**
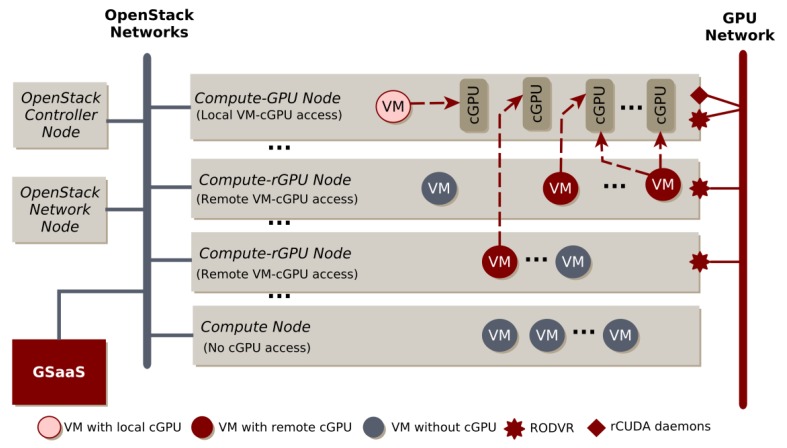
Components of GSaaS [72].

Taking the advantages of rCUDA, the authors of [71] discuss the implementation of an extension module to OpenStack, which enables configuring virtual machines initialized by OpenStack using remote GPUs. In [72], a GPU Scheduler as a Service (GSaaS) extension is implemented for OpenStack, based on rCUDA. GSaaS lets the user to define requests related to the needed GPU resources and scales the related resources according to the given requests and enables four working modes, including exclusive or shared using of local and remote GPUs. The components of GSaaS can be seen in Figure 8.

Each of these solutions can benefit from GPUDirect RDMA, which provides a direct access to the network device to the GPU’s memory therefore saving memory copying cycles.

### 6.4. High Performance Virtualized Networks

However, not only the physical devices are going through improvement steps, but the virtualized networks of cloud infrastructures do as well.

The authors of [73] show the implementation and performance of a virtualized underlay RDMA based network, and its performance. The relevance of such an overlay network can be seen when applications are able to use RDMA in a baremetal environment running in containerized environments, therefore the authors implemented a library that shows the verbs API used for RDMA, and can be used by the virtualized applications. The performance of the solution is almost identical to the baseline performance of native RDMA.

The authors of [74] introduce an SDN software switch prototype—ESwitch—that can easily scale over 100 Gbps. On the other hand, the authors give a detailed insight how Open vSwitch, the most commonly used open source software based switch, works. The paper also compares the prototype with OVS and shows that the ESwitch greatly outperforms OVS. The implementation of ESwitch relies on DPDK, as well as OVS was equipped with DPDK datapaths for the measurements.

### 6.5. Lessons Learned

There are more and more mature solutions for serving the increasing demands for reliable network architectures that support low latency communication and high bandwidth at the same time. On the other hand, taking the burden off the host CPUs FPGAs and smart NICs are getting evolved. For the effective processing of large amount of data, GPUs are utilized in clouds. However, the scheduling of these resources is crucial, therefore several techniques have been developed to share these resources in large scale cloud systems. In Table 4 and Table 5, we show the use-cases of the reviewed technologies.

### 6.6. Identified Research Gaps

Each of the reviewed technologies shows great development within the domain; however, we could not find research efforts that investigate the combination of physical network solutions, especially the combination of the two basic high performance networking solutions, DPDK and RDMA. GPU utilization is a hot topic nowadays, however, there is no public research activity to be found in heterogeneous GPU clusters. On the other hand, the diversity of accelerated network solutions limits the standardization of linking virtualized environments to the underlying high performance network.

## 7. Virtualization Technologies

When VNF/NFV technologies are chosen for the specific task, the underlying technology is resource virtualization.

The evolution of virtualization technologies shows a trend, according to which virtual environments are getting more and more lightweight. In the beginning, beefy virtual machines (VMs) had been running in the cloud using an own kernel; therefore, in the vast majority of the cases, running a full replica of the host operating system. However, the lightweight container based virtualization technologies provide less resource usage, although according to [75] containers provide less isolation than VMs. Unikernels (specialized, single-address-space machine images, also called as micro VMs) support faster startup times than containers and according to the measurements, unikernels offer very similar memory and CPU footprints to container-based solutions.

### 7.1. Function as a Service

Not only the technologies has evolved, but their use as well. Function as a Service (FaaS) is a new paradigm in cloud computing. FaaS supports virtualizing only building blocks of a higher level service in contrast with previous solutions where a complete service was encapsulated into a virtualized environment. FaaS lets the developers implement highly distributed applications, and can lead to more granular scaling strategies. The users of FaaS are only responsible to writing their functions and declaring when these functions should be triggered. The billing for FaaS is granular, as the user only needs to pay for the resources used during the function’s execution time.

FaaS is often linked with serverless computing that refers to a model where the existence of servers is simply hidden from developers, i.e., even though servers still exist, developers are relieved from managing and operating them. Serverless computing encourages and simplifies developing microservice-oriented solutions in order to decompose complex applications into small and independent modules that can be easily exchanged.

### 7.2. The Challenges and Solutions Related to Cold Start

Functions in such systems are running in a lightweight virtualized environment leveraging its low startup times. FaaS functions are short living, after the first invocation function instances are kept running by a short period of time; if no reinvocation happens during this period of time, the framework evicts them. However, the invocation of an evicted function takes much more time as the environment, in which the function is running, should be initialized. This phenomenon is called the cold start. Cold start is one of the challenges of FaaS, which can be mitigated by using unikernels instead of container based solutions [75]. The authors of [76] on the other hand show solutions to decrease the startup time of container based solutions. The authors also made analysis on the performance impact of using several Python libraries and implemented a solution to spectacularly decrease the latency and enhance the throughput of their solution compared to a Docker-based setup.

The importance of FaaS can be seen by the fact that besides several cloud service providers has their FaaS solutions, e.g., AWS Lambda, Google Functions, Azure Functions, the open-source community has also embraced this technology.

Several FaaS initiatives can be found on GitHub. A comprehensive overview of open-source FaaS systems is described by [77]. The authors of [78] examine the performance of OpenFaaS function runtimes in case of echo, compute intensive, and IO bound functions for Python, Node.js and Go. Furthermore the authors give a brief introduction to OpenFaaS and the examined language runtimes as well.

### 7.3. IoT and FaaS

A serverless or FaaS cloud platform can execute functions in response to events in addition to direct invocations. They enable rapid development by loosely-coupled event-driven programming paradigm that are a natural fit for many IoT applications, as IoT applications are often event-driven (devices trigger computation, communication, and storage events), have unpredictable execution profiles (requiring efficient autoscaling), and perform a variety of data processing and analytic tasks.

### 7.4. Considerations for Stateless Functions

FaaS is a promising solution for edge computing as it provides short living functions, therefore not occupying the limited resources of the edge infrastructure. On the other hand, FaaS follows the stateless programming model as functions in such systems are evicted after a period of time. Therefore, an external data-store should be in use, which poses new challenges. In a distributed system, data locality can highly impact the execution time of functions. The authors of [79] present the design principles of a low latency in-memory data store using RDMA. In [80], the implementation of DAL, a highly distributed data-store is detailed which supports data access in the orders of magnitude of microseconds. DAL is a distributed data-store supporting local and remote operations. For remote operations it supports both UDP and DPDK transport. Furthermore, this solution implements localization optimization by moving the data close to the software components which are using it.

### 7.5. Lessons Learned

FaaS is a novel approach to implement highly distributed applications by instantiating only small building blocks of a whole service. This leads to more mature scaling mechanisms. As functions in such a system are short living, FaaS technologies can easily be coupled with edge computing, where resources are limited. However, FaaS poses a lot of challenges as well. As a service in FaaS is implemented by numerous short living stateless functions, data should be moved close to the consuming function instances, on the other hand the startup time of function instances is also crucial in such systems. In Table 6, we summarize the features of each virtualization technologies reviewed.

### 7.6. Identified Research Gaps

Virtualization technologies are going through a great evolution; however, there are still numerous open questions. The combination of different virtualization technologies has not been standardized yet. The mitigation of security issues related to container based virtualization technologies is also a hot topic, however we still ensure the security of the container based architecture by running them on top of VMs. We can find open questions related to migration between different systems, as well. Furthermore, issues related to FaaS can also be found, as different FaaS implementations should be programmed in a different way, therefore a valuable effort would be the implementation of unified methods and APIs.

## 8. IoT Security in 5G

### 8.1. Security Requirements of IoT

Dealing with security concerns has always been a main challenge to solve in the field of IoT. Nowadays, the importance of handling privacy and security issues can not be overstressed, as the number of IoT devices are growing rapidly not only in civil/residential sectors, but in the industrial sector, as well. As IoT is an implementation and integration of different technologies and infrastructures, all security challenges and threats of each network technologies are inherited by the IoT system that utilizes these technologies. This implies that all layers of an IoT architecture are exposed to security risks [81].

Moreover, IoT systems have unique characteristics that raise new types of security issues that do not exist in conventional networks. Devices that operate in the sensor/physical layer of an IoT system usually have limited computational power and low storage capacity; therefore, standard security solutions such as public key encryption and spread-spectrum techniques can not be applied [82] by all endpoints. Additionally, these systems often consist of heterogeneous subsystems that have different capabilities regarding defense mechanisms. In this case, the achievable security level in the whole system is determined by the most vulnerable node. Therefore the current paper of ours focuses mainly on low-powered nodes as the “weakest link” in terms of IIoT network security.

Traditionally, security requirements within IoT and especially in IIoT applications are classified into three main categories—often cited as CIA. These are (i) confidentiality, (ii) integrity, and (iii) availability. Due to arise of new security threats, the CIA triad became insufficient to address every new threats, so other requirements such as Trustworthiness and Privacy became more important factors in this topic; however, these objectives are prioritized differently in industrial and residential applications.

In spite of the emerging number of (Industrial) IoT applications, there is still no general standardized layered structure for IoT systems. In most cases, the common approach uses three layers: (i) Physical layer (often Sensor or Perception layer), (ii) Network layer, and (iii) Application layer. Some might even include a Data Processing Layer, especially for classifying issues that are closely related to cloud based services [83], such as in Table 7, which summarize security challenges in different layers of an IoT architecture.

### 8.2. Security Issues of a Layered IoT Architecture

The **Physical Layer** of an IoT system usually includes sensors, actuators and any other node that gathers information and transmits it into the Network layer. These building blocks of a system are the most vulnerable parts of the whole infrastructure, as they mostly operate in external and outdoor environments, which means that they can be accessed physically. These types of attacks are refereed to as tampering, which includes not only ruining the device physically, but modifying it, such as replacing its firmware [84]. Tampering also can be the first step of executing a Denial of Service (DoS) attack against other nodes in the network. Generally, DoS attacks are major threats in each layer of an IoT architecture, but in the physical layer, they can even target closed systems by jamming. However, there is no general solution to avoid DoS attacks [83], and although spread-spectrum techniques would be efficient defense mechanism, but they can not be widely applied due to the limited computation capability and power consumption [82].

The heterogeneity of devices in the **Network Layer** implies that the types of security threats and attacks vary in a wide range. Although endpoints in the cloud and in the edge are more powerful devices where traditional security solutions can be adopted, the majority of the nodes still lack of these features such as public key cryptography techniques [85]. Therefore, these nodes often limit the defensing capability of the whole network, while they are its weakest links. For example, compromising a node may win the trust from other devices, so the trustworthiness of the system can be ruined this way. However, there are indirect attack types that may be even more dangerous than the direct ones, as it is harder to discover them. A man-in-the-middle—as the most prevalent indirect attack type—can be more harmful to any IoT system by violating the confidentiality, integrity, and privacy of restricted data. Such an attack can be executed in different ways like eavesdropping the messages or replaying them, which violates privacy, but it can also deteriorate QoS by changing not encrypted routing information [86].

The **Application Layer** usually consists of different web applications, mainly interfaces, service management tools and middlewares [87], thus traditional software attacks mean the major security risk in this layer. For instance: insufficient validation of an input may enable injecting malicious input. As a consequence, attackers can perform various operations, depending on the software, such as stealing data, compromising database integrity, or bypassing authentication [88].

### 8.3. General Security Considerations within 5G

Since 5G was introduced, a huge emphasis is put on its benefits as an optimal telecommunication platform for IoT applications and systems. 5G meets such requirements related to IoT that can not be fulfilled before, in the terms of bitrate, latency, reliability, and many others. However, as general insufficiency of security in IoT systems remains unresolved, it is mandatory to discuss how security considerations have been taken into account in 5G in respect to IoT scenarios.

Being a wireless communication network, a significant part of security schemes and solutions applied in 5G are adopted from other wireless technologies, but as it uses licensed spectrum, the main security concerns is inherited from legacy cellular networks, such as its ancestor, Long Term Evolution (LTE). It is also beneficial that fundamental technical improvements that aimed to enhance efficiency are valuable techniques against numerous security threats, such as Massive MIMO against eavesdropping. However, applying legacy security solutions, besides many others, plays only a minor role of the whole scheme, as security is intrinsically an integral part of the overall architecture. Therefore, it has flexible security scheme and methods by design to (i) deal with new requirements derived from huge number of possible use-cases where 5G will be utilized, (ii) support new trust models as well as new threat-handling models, and (iii) handle MMTC traffic and management – massive number of various types of connected devices.

Before discussing concrete proposed solutions, it is worth reviewing what general considerations does 5G have regarding traditional CIA requirements of IoT security. Due to the separation of control and user planes, networking mechanisms are implemented in a different way than previous cellular networks: most functions are virtualized now, so they are purely software. Therefore, technologies, such as SDN and NFV, play a major role in implementing network elements and functions to provide the aforementioned high flexibility as well as network slicing. Using these technologies makes it possible to apply different approaches and techniques for each individual use case, thus adjusting 5G networks to specific criteria. On the other hand, all of these new solutions bring new security threats and challenges into 5G based systems [89]. Table 8 provides a summary of security challenges regarding technologies that 5G is build upon. Therefore, standards and related researches focus almost exclusively on solving these 5G specific issues, instead of use-case specific ones, i.e., CIA in an IIoT manner.

The most important threats are well-known, such as the aforementioned DoS and man-in-the-middle attacks, where their main targets are SDN- and NFV-based elements, so the core parts of the network [90]. Although these are general 5G issues and not IoT-specific, it is still worth mentioning that the control plane in 5G relies upon cloud is basically operates as a cloud, thus general 5G threats can be considered as IoT related issues.

Otherwise, there are numerous proposed techniques and technologies exist to enhance security features in the 5G infrastructure, although most of them are not applicable for IoT systems, including really low-powered nodes and sensors. The following ones are related to certain requirements of IoT and even more so of IIoT, and they can, with minor restrictions, be applied in 5G networks [91].

### 8.4. Low-Powered Security Enhancements in 5G

Although authentication is traditionally not a part of the CIA triad, it plays an important role in fulfilling these requirements. Authentication scheme has also improved and been more flexible in 5G networks, mostly due to low-latency requirements. To meet low latency needs, an SDN-based fast authentication scheme can be used that relies upon weighed secure-context-information. After a full (and more computation intensive) authentication, user-inherent physical layer attributes are used for identification to provide seamless handover authentication between cells, or even trusted parties [92,93].

As 5G applications can transmit massive private and/or critical data, **confidentiality** has to be ensured in these use-cases. 5G puts a lot of emphasis on Physical Layer Security (PLS) solutions as well, thus instead of relying upon only high-level data encryption methods, PLS support can enhance defense mechanisms in this field (i.e., against jamming). There are multiple proposed techniques such as power control, artificial noise, and multi-level cryptography methods; however, most of them require more computation capability than a typical IoT node has. Relaying is considered as a lightweight solution to ensure certain level of data confidentiality in 5G for IoT applications [93]. Additionally, there are numerous lightweight cryptography methods that can be applied [94]; however, there is no standard about which one should be used, since it greatly depends on the use-cases.

**Availability** issues—in wireless networks these are related to DoS attacks and performed by jamming in most cases. As we mentioned before, using spread-spectrum techniques is an efficient way to avoid jamming, although they can not be applied in IoT nodes with limited resources (e.g., sensor). In this field 5G does not deploy revolutionary new solutions—besides a few ones—although Labib et al. in [95] proposed a solution where a fusion center defenses nodes from jamming attacks over a 5G network.

### 8.5. Lessons Learned

**Figure 9:**
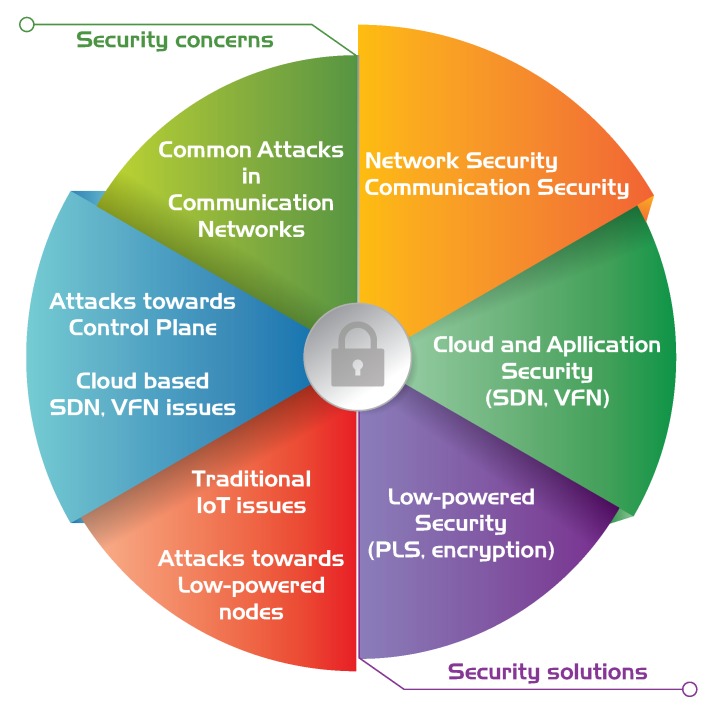
Security concerns (left) and solutions (right) in 5G-based IoT networks.

5G offers various benefits for IoT and IIoT systems, including low latency, high speed, and many others, but it also involves its own security issues. These are mainly related to the nature of communication itself and the SDN/VNF-based network elements on the control plane. 5G-supported IoT is a hot topic now, therefore the number of proposed solutions are emerging, which enables 5G to be an optimal communication platform for IoT. Traditional security concerns in IoT networks and 5G are very common, thus they are addressed during the standardization of 5G; however, specific ones, such as in the case of low powered nodes and sensors, are not. The greatest advantage of 5G regarding IoT security is the flexibility of the system, thus efficient, use-case specific solutions can be applied. Regarding low-powered nodes, which are huge drivers for IoT, using PLS solutions and lightweight cryptography techniques is an effective way to improve security. The unified scheme for security of 5G based IoT networks is shown in Figure 9.

### 8.6. Identified Research Gaps

Security has always provided infinite research material in a wide variety of topics. Nowadays, cloud security is already one of the hottest research fields, partly because of 5G and IoT and partly on its own. In 5G, it has an important role on application side as well as on control plane of which defense is critical task.

On the other hand, device security, especially in low-powered node is sort of a well-known research field. It has many proposed solutions which address defending hardware and software of these type of nodes. PLS solutions in 5G are really promising as well; however, most of them are not implemented or tested in real-life scenarios or use-cases, therefore almost none of them applied in practice. As the lack of the aforementioned scenarios, it is highly desired to apply them in real use-cases, as well as measuring their performance and reliability or comparing these technologies to each other.

## 9. IoT and Smart Contracts on Distributed Ledgers

### 9.1. Motivations of Blockchain Utilization in Industrial IoT

Industrial IoT has extra concerns on data security and privacy, as discussed earlier. This is especially true within multi-stakeholder environments where parties do not necessarily trust each other, but are eager to have business relationships. Production-focused sectors of the industry tend to build on established, safe, and secure methods of info-communication technologies; as both introduction and the in-production error correction has high stakes and price.

The technical requirements of real-time operation, security and safety, high reliability, complexity, engineering efficiency are already available for the early adopters of IIoT solutions. On the other hand, non-technical aspects, such as return of investment and trust are hard to justify when introducing new technologies with such a great paradigm shift as digitization for Industry4.0. Although trust among new partners is built through the involvement of legal and financial authorities, this do not cover end-to-end supply chains—just overlapping ecosystems. This is a relatively slow, but seemingly necessary process, which, again, will not cover trust among all parties in the supply chain, just those who interact. This also means that stakeholders at the end of the logistic chain have no easy way to go after initial vendors, their procedures, not to mention their failures.

The CIA triad can be further enforced by using blockchain technologies: confidentiality is provided by the Public Key Infrastructure (and methods alike), Integrity is a core concept ensured by the immutable feature, whereas Availability is there due to massive distribution of the ledger. Furthermore, traceability and reliability of IoT data are also inherited by the immutable distributed ledgers—so time-stamped data inclusion and modification can be traced back, and data quality of the data remains trustworthy [96].

The immutable feature of distributed ledgers, and their verification through various blockchain technology methods make it possible to leave out third parties—central authorities—who are involved merely to build trust. Together with smart service contracts, the use of private blockchains within industrial partnerships or ecosystems make transactions fast, secure, safe, trusted, validated and immutable throughout the various supply chains.

### 9.2. When to Use Blockchains for IIoT?

Use-cases where even per-item micro-transactions should be noted and acknowledged, or where various parties of the supply chain should access validated data on the smart product (footprint), the data storage on a distributed, replicated ledger makes sense. Furthermore, for transactions (either sensor data or actual physical equipment) on a highly changing market with various vendors who are not always trusted, blockchains powered with smart service contracts can enforce contractual steps. These are potential applications of blockchains in an IIoT environment.

**Figure 10:**
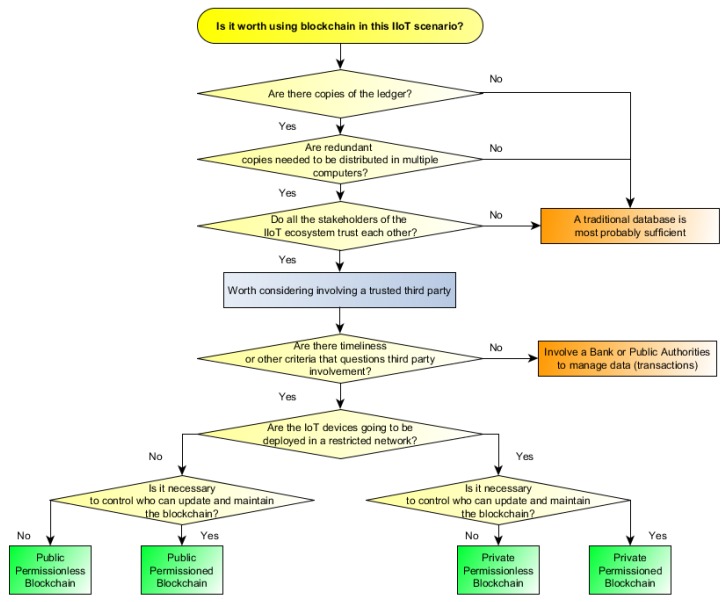
Decision support on considering the use of blockchains in the given IIoT scenario.

On the other hand, in many cases, it is not justifiable to use distributed ledgers or blockchains for industrial IoT scenarios. When specifically taking the data storage, access, security, and privacy issues, Figure 10 provides a decision support flowchart whether it is worth considering the usage of blockchain technologies for the given IIoT scenario. This flowchart was strongly motivated by the work in [97,98].

Current blockchain systems suffer from several drawbacks when considered for IIoT scenarios: the currently achieved number of transactions (less than 100 per second) can be slow for many applications; the mixed use of private & public blockchains is not clearly solved; vulnerabilities such as the infamous 51% attack, race attacks, finney attacks, and the fact that bugs in smart contracts are not patchable, are a few of the more severe ones.

Advantages and drawbacks of using blockchain technologies for IoT are discussed by [98,99,100]. We summarized the main findings in Table 9.

### 9.3. A Brief Survey on Using Blockchain Technologies for IIoT

There are already several survey papers available on the utilization of blockchain technologies for IoT [98,101], describing use-cases and current research trends for blockchains concepts that may fulfill IoT requirements.

Turning towards more industrial applications, the crucial blockchain platform requirements for IIoT are analyzed by [102], addressing issues of security, fault tolerance, durability, Public Access possibility, consensus algorithms and smart contract types. The same authors describe various actual blockchain implementations in [103], which are differing in block creation, consensus mechanisms and smart contracts support. Regarding IIoT and blockchain integration they propose that a new module should be created that connects the two technologies.

From the Industry 4.0 and IIoT application aspect, the authors of [104] survey various case studies, covering the power industry, production/manufacturing, supply chain, logistics, agriculture, healthcare and the retail industry. They compare the proposed solutions through platforms and consensus algorithms used, data transferred, and whether it utilizes smart contracts or not.

Related to 5G-enabled automation IoT, the authors of [99] provide a systematic review on solutions and challenges for blockchain technology utilization. The discussion within this paper is divided into three parts, firstly the background of blockchain, IoT, and 5G are covered, briefly followed by respective industrial applications, then lastly, as the main points, open issues and challenges are covered mainly for industrial applications. The paper covers blockchain applications at the domains smart homes, smart cities, healthcare, agriculture, Industry 4.0, supply chain management, as well as autonomous vehicles, unmanned aerial vehicles, and multimedia and digital rights management.

The authors of [105] focus specifically on electric vehicle cloud and edge (EVCE) applications of blockchain technologies, whereas [106] provides a joint cloud study on smart travelling.

Without aiming for another comprehensive survey of the field, let us merely highlight two typical approaches for lightweight blockchain proposals intending to fit IIoT needs. The BPIIoT platform [107] targets to to support the development of decentralized, end-to-end manufacturing applications, and uses a blockchain network powered with smart contracts serve as an agreement between service consumers and manufacturing resources to provide on-demand manufacturing services. In order to reduce the load and delay of the network, the BPIIoT platform is designed as a light-weighted network architecture consisting of an on-chain network and an off-chain network. All transactions are carried out on the on-chain network, such as digital signature based on admission control, programmable licenses, etc., the off-chain network handles problems that cannot be solved by blockchain technology, such as storage, complex data processing, and so on. Another typical approach is to decrease the computational burden of resource-constrained nodes, as the authors of [108] propose. They selected three lightweight hash functions (QUARK, PHOTON, and SPONGENT), which show better performance in perspective of implementing area, throughput, and power consumption. These hash functions can ensure cryptographic security and be implemented with minimal efforts for resource-constrained devices. Then, using these hash functions, they connected each data block by flexible hashchain. This approach can reduce the computational burden and latency. In order to improve scalability of the network, this solution introduces area control nodes that control all nodes in a given area or cell.

Motivated by increased information security needs for IIoT-systems organized in wireless sensor networks, mechanisms from blockchains and distributed ledger technologies were derived within [109], and adopted to microcontrollers, with a small energy budget and low calculation capabilities. The authors argue that principles such as chained blocks, distributed ledger, time-stamping and consensus could actually be transferred, which is a threat. This leads to a higher effort for intruders to gain access to the communication process and to inject false information.

### 9.4. Lessons Learned

Distributed ledgers, Smart Service Contracts, and other blockchain-related technologies can be beneficial to be used in certain IIoT scenarios, although their introduction must be supported by hart-to-make decisions, as Figure 10 and Table 9 show.

While the current chapter merely provides an overview on the above issues and solutions, readers are encouraged to study the current surveys on the details of IoT blockchain technologies [96] as well as their comprehensive comparisons and applications [110]. Based on the already enormous amount of survey papers that discuss application areas as well as technological improvements, Blockchain IIoT is arguably an area with very current, theoretical and applied research initiatives.

### 9.5. Identified Research Gaps

Industrial IoT has a high potential for using Smart Service Contracts, Distributed Ledgers, and other blockchain-related technologies. The main research gaps of this area are the following:

- increasing the transaction rate for well over 1000 transactions per second;
- providing reliable and secure interoperability between designated public and private clouds;
- providing architectural means for resource constrained IoT devices to utilize the related technologies as much as possible;
- allowing secure and trusted ways to correct unintended errors in smart service contracts;
- clarification of hidden costs when using blockchains for IIoT to allow objective comparisons with traditional alternatives.

## 10. AI Support for 5G Operations and Services

5G systems have brought us the long-waited promise of support for Enhanced Mobile Broadband (eMBB) services in addition to the introduction of machine-type devices, interconnected through the Internet of Things for massive Machine Type Communications (mMTC), as well as novel services that require Ultra-Reliable Low-Latency Communication (URLLC). The emergence of this range of innovative mobile applications and online services pose unprecedented challenges to the fifth generation of mobile networks, which are thus required to introduce new solutions and mechanisms to optimise the use of limited resources, e.g., finite radio spectrum. Moreover, compared to existing mobile communication techniques, 5G has more varied applications, thus its corresponding system design is more complicated. The resurgence of AI techniques offers an alternative option that is possibly superior to traditional ideas and performance of system management.

In addition to the new requirements of increased network capacity, reliability and latency, future networks are still expected to meet requirements of energy efficiency and heterogeneity of user terminals and Quality of Service/Experience (QoS/QoE) demands. In a concept paper [111] the authors outlined the AI challenges which need to be overcome to enable the perception of zero delay networks. Typical and potential research directions related to the promising contributions that can be achieved through AI were identified, evaluated, and investigated in [112] that combed through several promising research directions in AI for 5G technologies based on an understanding of the key technologies in 5G. The authors of [113] surveyed how 5G network management, with an end-to-end perspective of the network, can significantly benefit from AI/ML solutions. The authors reviewed and provided the basic concepts and taxonomy for SON, network management and ML; and also analyzed the state of the art in the literature, standardization, and in the market.

Based on these recent surveys taxonomies, we group the use cases of AI technologies in the 5G ecosystem as follows.

- Service management: operations support systems, resource provisioning, fault localization, failure root cause analysis, business support systems, security;
- Network and cloud resource management: flexible function deployment, NFV orchestration, network slicing, green operation;
- Radio management: air interface coordination, site collaboration, user mobility.

### 10.1. Service Management

More intelligence built in to the 5G system will allow for a shift from managing networks to managing services. Intelligent functions can be customized for each of these services allowing them to operate more resiliently and securely, taking the mobile network to a new level of innovation for the benefit of industry and society [114]. While digital transformation accelerates service innovation, it also requires automation abilities, as mobile operators need a much more flexible network to satisfy the increasing demands of the new services [115].

Already in 2014, Ref. [116] showed the effectiveness of proactive caching, a service-level management mechanism proposed for 5G systems, in two case studies that exploited the spatial and social structure of the network. First, in order to alleviate backhaul congestion, they proposed a mechanism whereby files are proactively cached during off-peak periods based on file popularity and correlations among user and file patterns. Second, leveraging social networks and D2D communications, they proposed a procedure that exploits the social structure of the network by predicting the set of influential users to (proactively) cache strategic contents and disseminate them to their social ties via D2D communications.

The authors of [117] presented the limitations of the current network and service management and described in detail the challenges that 5G is expected to face from a management perspective. They presented a set of use cases and scenarios of 5G in which machine learning can aid in addressing their management challenges. They argued that machine learning could provide a higher and more intelligent level of monitoring and management of networks and applications, improve operational efficiencies and facilitate the requirements of the future 5G network.

In [118] the authors proposed an intelligent IDS taking the advances in software-defined technology and artificial intelligence based on software-defined 5G architecture. The solution flexibly integrates security function modules which are adaptively invoked under centralised management and control with a global view. It can also deal with unknown intrusions by using machine learning algorithms. Evaluation results proved that the intelligent IDS achieved good performance with low overhead, and the selected machine learning algorithms showed good accuracy in flow-based classification.

The authors of [119] proposed a novel 5G-oriented cyberdefense architecture to identify cyberthreats in 5G mobile networks efficient and quickly enough. For this, their architecture used deep learning techniques to analyze network traffic by extracting features from network flows. Moreover, their proposal allowed adapting, automatically, the configuration of the cyberdefense architecture in order to manage traffic fluctuation, aiming both to optimize the computing resources needed in each particular moment and to fine tune the behavior and the performance of analysis and detection processes.

The application possibilities of deep learning in mobile telecommunications network and service management has first been summarized by [120], as shown in Table 10. The FCAPS represent Fault Configuration, Accounting, Performance and Security Management respectively—and these are matched with deep learning target applications, such as prediction, anomaly detection, clustering, or classification. Besides, the authors demonstrated a use-case where they manage to predict call-establishment errors to happen within the IP Multimedia Subsystem (IMS) based on previously learned correlations with delays on certain network paths.

### 10.2. Network and Cloud Resource Management

5G networks are likely to co-exist with 2G/3G/4G for the foreseeable future, which will bring a significant challenge in operating a 5G network, especially about the traditional operating model [115]. Namely, the coexistence of 2G, 3G, 4G and 5G networks brings difficulties in network synergy and interoperability, difficulties in fault demarcation and location under the hierarchical decoupling architecture, and also challenges of unified resource scheduling and operations due to the dynamic change of cloud and virtual networks. Therefore, the challenges of network operations that come with the 5G era will be significant.

The advanced automated operations are gradually forming a gap with the traditional operations based on expert experience. Automated and intelligent network operations will be the just need in the 5G era. AI technology has intrinsic advantages in solving high-volume data analysis, cross-domain feature mining, and dynamic strategy generation, and will create new modes and capabilities for 5G network operations [121]. The mobile network automation will rely on automatic closed-loop under the operator provided intent or policy [115].

In [122] the authors proposed AI as a built-in architectural feature that allows the exploitation of the resource elasticity of a 5G network. Building on the work of the recently formed Experiential Network Intelligence (ENI) industry specification group of ETSI to embed an AI engine in the network, they described a novel taxonomy for learning mechanisms that target exploiting the elasticity of the network as well as three different resource elastic use cases leveraging AI.

### 10.3. Radio Management

The rudimentary concepts of machine learning are reviewed in [123] and the authors proposed ML employment in the compelling applications of 5G networks, including cognitive radios, massive MIMOs, femto/small cells, heterogeneous networks, smart grid, energy harvesting, device-to-device communications, and so on.

In [124] the authors proposed an efficient online Channel State Information (CSI) prediction scheme for predicting CSI from historical data in 5G wireless communication systems. Specifically, they first identified several important features affecting the CSI of a radio link and a data sample consisted of the information of these features and the CSI. They designed a learning framework that was a combination of a CNN (convolutional neural network) and an LSTM (long short term with memory) network.

A deep learning-based pilot assignment scheme (DL-PAS) for a massive MIMO system is proposed in [125]; the system utilized a large number of antennas for multiple users. The proposed DL-PAS improved the performance in cellular networks with severe pilot contamination by learning the relationship between pilot assignment and the users’ location pattern. The authors designed a novel supervised learning method, where input features and output labels were users’ locations in all cells and pilot assignments, respectively. The proposed DL-PAS provided a near-optimal pilot assignment from the produced inferred function by analyzing the training data.

In [126], an interference-aware path planning scheme for a network of cellular-connected unmanned aerial vehicles (UAVs) was proposed. In particular, each UAV aimed at achieving a tradeoff between maximizing energy efficiency and minimizing both wireless latency and the interference level caused on the ground network along its path. The problem was cast as a dynamic game among UAVs. To solve this game, a deep reinforcement learning algorithm, based on echo state network (ESN) cells, was proposed. The introduced deep ESN architecture was trained to allow each UAV to map each observation of the network state to an action, with the goal of minimizing a sequence of time-dependent utility functions. Each UAV used ESN to learn its optimal path, transmission power level, and cell association vector at different locations along its path. The proposed algorithm was shown to reach a subgame perfect Nash equilibrium (SPNE) upon convergence.

### 10.4. Applied Artificial Intelligence Methods

#### Machine and Deep Learning

Statistical learning and symbolic reasoning have been developed largely by distinct research communities. Ericsson argues that hybrid approaches will be useful in intelligent 5G systems where robust learning of complex models is combined with symbolic logic that provides knowledge representation, reasoning and explanation facilities [114].

#### Real-Time Analytics

Real-time requirements entail that predictions, model updates and inferences from knowledge bases are based on live-streaming data. This has implications for system architecture – how to distribute the models and knowledge bases over the cloud, edge and devices; whether model training should be offline or online; how to represent and prepare data for fast consumption by algorithms, and more. The systems must support flexible, programmable data pipelines for the volume, velocity and variety of real-time data and algorithms capable of real-time decision making [114].

#### Distributed Learning Algorithms

There is a need to leverage the scale of distribution, make appropriate abstractions of local models and transfer the insight to other local models. Learning about global data patterns from multiple networked devices or nodes without access to the actual data is also possible. A recent approach is so called federated learning: learn local models based on local data patterns, send the local models to centralized cloud, average them and send back the average model to all devices [114].

### 10.5. Lessons Learned

The below Table 11 summarizes the areas of application of AI methods within 5G systems, and the related work touching upon different aspects of those areas.

### 10.6. Identified Research Gaps

While application-agnostic AI-based improvements are widely investigated in the 5G platform, there is room for advancements for smart solutions in the industrial IoT scene. AI-based optimization methods, specifically crafted for such applications running on 5G connectivity and compute capabilities, have the potential to ensure higher service reliability and more economical operation. For example, robot control on a factory floor can be assisted with intelligent forecast methods by learning reoccurring patterns, and making decisive actions before sensor-based reactive decisions would be made. Such time series analysis and recurrent neural network learning algorithms should be implemented in the 5G infrastructure’s edge to keep latency to the least possible level, and crafted carefully to the needs of the IoT application. The research gaps are therefore found to lie within the IoT application-specific information services that the 5G platform could offer. AI-related platform APIs that provide management services as the ones summarized in Table 10, could enable application programmers to integrate their IoT systems fast and seamlessly to 5G providers’ offerings.

## 11. Discussion on Private Campus Networks

### 11.1. Service Providers for the Customers

When 3GPP designed the services and defined the areas to be covered by 5G, three basic theoretical directions and objectives were identified [127]. As part of 3GPP, which has been entrusted with the development of 5G service level systems, IMT-2020 [2,128] has set out its goals as follows.

- Innovation—Deploy innovative software-based services for both the enterprises and the individual subscribers. Innovation must mean software based services, where innovation serves not only individual subscribers but also corporate customers. Therefore, a particular function or service must be freely transferable between the physical elements of the network and hardware.
- Digitalization—Service Provider (SP) must recognize that 5G has a shift towards cloud services. SPs need to be clear that the next step in digitalization is moving towards cloud services. Therefore, it is not preferred to bond services to a specific hardware. They must be deployed on various cloud infrastructures. Therefore, these services can be customized freely and flexible.
- Simplification—Want to Virtualize (Network Function), automate, and simplify. When designing the system architecture [129], it must be initially taken into consideration that elements can be developed (I), designed (II), and operated (III) by machines. The system must be built of simple and interconnected elements which form a loosely coupled digital system. The base elements of the services should have minimal and loose connections; also, the associated services should be modular.

Furthermore, support for legacy systems is a practical addition: customers with less needs and efforts must be served as well as new customers. Service Providers shall pay attention to legacy customers’ needs [130]. In case some customers require merely voice services, providers shall respect this and fulfill their needs. This also means that new services shall be at least as simple as existing ones.

The following subsections provide various hints for motivating private campus networks, where enterprises can utilize complete 5G network segments as they would own it (depending on certain regulations they could even own it) or experience network and service quality as they were the only users of the network. This motivation is built up by a short recap on the evolution of mobile cellular communication and services, and the expected business impact of 5G.

### 11.2. Mobile Evolution

Generally, in the mobile industry, the promises of generation “N” are fulfilled by the “N+1” generation in practice. The main use-case for “1G” phones was the transmission of the human voice with full mobility of users. The service providers only offered services with large size User Equipment (UE) where cell-to-cell handovers were not fully seamless. Thanks to the growing interest in this technology and the rapidly growing subscriber numbers, standardization committees have developed a system that provides a globally available voice transmission service, Global System for Mobile Communications (GSM; as later known as “2G”). Using the GSM standard, the UEs were able to move between cellular towers without interrupting duplex voice traffic; also, the devices could be used in other countries as well. However, data transfer was only added later, so the data transmission rate was quite low. The 384 kbps throughput and ~200 ms one-way latency available in Enhanced Data for GSM Evolution (EDGE) provided a basic data network service, but could not at all compete with the current Digital Subscriber Line (DSL) or Cable Television (CaTV) data transmission.

As service providers have seen that mobile data could have a bright future, it became the primary focus for their research and development. The voice transmission on the 2G network was almost perfect, but the growing number of users needed better spectral utilization. The 3G standard is already partly designed for data transmission along with the voice. The transfer rate was up to 42 Mbps. However, at the time of writing the standard, the main use-cases were known as web browsing and huge file downloads only. At that time, “killer applications” such as Facebook and Youtube were not particularly known, although they required much more data and continuous data transfer from the network site. It is clear that loading a web page has different demands on the network side than a continuous chat, even with Rich Communication Services (RCS) features, including eventual video messaging. To cover these diverse requirements, the 4G network was standardized to provide dynamic bandwidth allocation and serve the need of many users at the same time. The targeted peak data rate has ended up being 1 Gbps—with major question marks whether this has been reached by 4G in practice.

The major plans for 5G systems around 2020 contain even higher data transfer rates (10 Gbps), more interactive services (1 ms latency), and more users (0.5M users/km^2^). The network is slowly becoming live and fulfilling the criteria described, but for now, the actual major use-cases, “killer applications” are highly awaited for 5G. As an example, the 1 Gbps data throughput cannot be leveraged by today’s smartphones compared to 4G’s 750 Mbps, so the user experience is not significantly better. Therefore, the extra infrastructure investment would not pay off in the short-term for this use-case.

### 11.3. Business Impact of 5G

5G’s new use-cases are generating tension currently for service providers, device manufacturers, and the industry in general. It is clear that 2G-3G and 4G networks are designed primarily for people in terms of use-cases. Of course, a significant number of machines are connected to these networks as end-devices, as well. The almost untapped resources to be provided by 5G are likely to be utilized by machines. These use-cases can be the simplest household appliances, vehicles, and of course complex industrial robots [131]. Hard real-time connectivity=–when a sensor-to-machine delay is less than 1 ms—will be utilized by several use-cases, however mostly not human applications [5,26]. With 5G, we will be able to achieve a real-time production monitoring, knowing the current state of equipment we are ordering, or the time when it is expected to be manufactured, with proper access rights from anywhere around the globe. It also facilitates the development of real-time business and the advancement of services where precision has immense business importance. All in all, this means that the spread of 5G will benefit the economy as a whole [131].

To understand the exact economic effects, we must examine which parts of the industry are affected and to what extent. There are many studies for this phenomenon, certainly updated or revised year by year. According to a study published by Ericsson, it is estimated that 5G-enabled industry digitization revenue for ICT players will reach $ 1.3 Trillion in 2026 [132]. When examining the most prominent domains, energy and utilities account for 19%, industrial production are there for 18%, and it is worth highlighting the automotive industry, which accounts for 8%. In terms of industrial networks, naturally, the factories are the main stakeholders. The short term impact of 5G and IoT is expected between $1.2 and $3.7 trillion. Of course this includes other high-impact domains utilizing 5G, such as connected cities and vehicles [133].

### 11.4. Private Campus Network—New Possibilities for Industry 4.0

The arrival and development of Industry 4.0 represent a revival similar to previous industrial revolutions in history [134]. The revolution in Industry 1.0 was the using complex machines to make work easier and faster instead of manual efforts. The main invention of the first industrial revolution of 1784 was steam, and chain-driven equipment such as the weaving machine. The second industrial revolution took place in the 1870s when mass production first appeared, developing techniques for mass production and enabler functions, such as assembly on a conveyor belt, or whether a workflow is always done by one person or a small group of the same people. The third industrial revolution dates back to 1969, when microelectronics brought a new phase of technology. Based on the program code, a robot/machine was able to perform several operations and work phases replacing complex manual work. The program was loaded only once and was executed, based on various incoming information very precisely. The fourth industrial revolution is underway, with production lines and robots becoming more intelligent. We are able to create robots that download and use the program, which means that the same production line can adapt to an industrial need very quickly [4]. As a high-level example, at one minute, the workstation produces a Type A car, and in the other one it can create a Type B car. To accomplish this, all equipment needs to be involved in the reconfiguration of the manufacturing process and must be connected as cyberphysical systems.

To fulfill the requirements of Industry 4.0 criteria and use-cases, there is a need for a unique network, which may be a cellular network with strict service guarantees [3,5], either part of the global/national infrastructure, or a private campus network for enterprise purposes. As the use-cases evolve, there is a need for system evolution and continuous improvement. Continuous feedback on how well the developed service meets the expectations and user-needs is also valuable. Based on these considerations, the network solution could have the following characteristics:

- A solution based on recent standards: The new 5G standards and architectures provide the opportunity to build a highly flexible network. Some services can run on a small portion of network elements. Therefore, we are able to utilize merely a small percentage of network resources in order to provide elementary service needs. This allows to design services and implementations to individual customer needs. With the interoperability of standards and interfaces, systems will be able to co-operate and provide high-quality experience.
- Unlimited data consumption: Industrial equipment will have no other connection to the IP services world than 5G, so they need to have an unlimited data subscription package. Of course, these resources will be available at a limited data rate, uniquely for every use-case.
- Dedicated Radio, Core, IP resources for a given use-case: For these needs, it is clear that dedicated radio, core network elements, and IP resources will be required. Dedicated items mean, among other things, that the services can be used exclusively by those devices that authorized on this network.
- QoS for service separation with Service Level Agreement: Guaranteed SLAs for use-cases must be provided in order to receive the appropriate QoS. It is expected that a robot communicates continuously. Therefore, the Busy-Hour interpretation, which is based on the daily routines of human cycles, is unlikely to be present in case of machine type communication. Thus, when calculating SLA, QoS and capacity management, it must be taken into consideration that all devices are continuously using the allocated resources.
- Goals: A planned private campus network can be considered complete if it fulfills the goals set by the use-cases and can ensure them continuously. The fundamental parameters are throughput, latency, and high transmission rate together with extremely high reliability and availability (99.999% to 99.999999%). On the other hand, in the current state of industrial networks, these have not been well defined yet.

### 11.5. Research Gaps for Private Campus Networks

- Radio Access Network (RAN): Publicly available research material contain very little information on how to cover an industrial area by radio. Although there are several kinds of research on WiFi coverage in industrial areas, unfortunately, these researches hardly include any case studies on cellular coverage. Measurements in the industrial sited, under live conditions, provide great feedback to standardization bodies [135]. It is clear that in a manufacturer’s environment, different drilling, cutting, pressing and welding machines operate, and radio interference is difficult to model accurately through theoretical methods. In order to get proper base-date, multi-directional measurement execution and analysis must be carried out. Furthermore, the appearance of TSN [136] is an exciting direction, but further investigation is needed on its practical, scalable feasibility.
- Network slicing: Although the theory of network slicing is vast, there is very little information available at this stage on real implementation and feedback from industry. It is necessary to examine how the terminals and standards work and whether they meet the needs of the industrial sector.
- Core network orchestration: It is clear that systems and architectures need to be designed to the need of the industrial enterprise in question. To do this, the mapping of the transmission technology parameters are needed first, which are mostly unknown to the customer, and then build a corresponding network. Although theoretical studies of such methods already exist, detailed elaboration is required.
- Network and service monitoring: The life cycle of industrial mobile networks is unknown [131]. Private campus networks are to be operated in a relatively closed and secured manner, although end-devices has to be made accessible for networking personnel for supervision. The human in the loop appears here, and it is crucial to have a deeper understanding of procedures and task to be covered here, together with the exploration of the proactive maintenance possibilities in this area.

## 12. Conclusions: Consolidated Challenges and Research Gaps

The major challenges regarding 5G support for industrial IoT can be grouped into the following categories.

- proving that QoS metrics meet requirements in real-life: latency, jitter, loss, throughput, and availability, and the reliability of these metrics;
- interoperability of various network elements and segments; conformance testing;
- meeting security requirements and addressing privacy concerns;
- scalability: meeting high density, mass IoT, and URLLC criteria together;
- providing effective device-to-device solutions not only device-to-cloud;
- exploiting the capabilities of Multi-access Edge Compution, in other words, the mobile edge-cloud;
- effective and economical NFV and SDN solutions;
- large scale deployments, network and endpoint operations and maintenance;
- standardization to be fast, and creating firm interfaces as well as allowing for evolution;
- utilizing emerging technologies, such as distributed ledgers and smart service contracts—for multi-stakeholder environments to ensure flexible, safe and secure data management;
- take advantage of AI technologies when addressing challenges of network, service and resource management issues;
- deployments of private campus networks.

Each Section of this paper has been concluded with “lessons learned” and “research gaps” to summarize the main take-away messages in relation to the given aspect of 5G-supported Industrial IoT challenges, solutions, and research gaps.

## Figures and Tables

**Table 1:** Data rate and density requirements for various broadband scenarios [3].

Scenario	Experienced Data Rate (UL and DL)	Area Traffic Capacity (UL and DL)	Overall User Density	UE Speed
Urban macro	25 Mbps 50 Mbps	50 Gbps/km^2^ 100 Gbps/km^2^	10,000/km^2^	Pedestrians and users in vehicles (up to 120 km/h)
Indoor hotspot	500 Mpbs 1 Gbps	2 Tbps/km^2^ 15 Tbps/km^2^	250,000/km^2^	Pedestrians
Broadband access in a crowd	50 Mbps 25 Mbps	7.5 Tbps/km^2^ 3.75 Tbps/km^2^	500,000/km^2^	Pedestrians
High-speed train	25 Mbps 50 Mbps	7.5 Gbps/train 15 Gbps/train	1000/train	Users in trains (up to 500 km/h)
High-speed vehicle	25 Mbps 50 Mbps	50 Gbps/km^2^ 100 Gbps/km^2^	4000/km^2^	Users in vehicles (up to 250 km/h)
Airplanes connectivity	7.5 Mbps 15 Mbps	600 Mbps/plane 1.2 Gbps/plane	400/plane	Users in airplanes (up to 1000 km/h)

**Table 2:** Use cases of 5G technologies and their realization.

	Number of Use Cases					
	**eMBB**	**URLLC**	**mMTC**	**Other**	**Realization**	**Summary**
[19]	1	1	1	mobility, reliability	– none –	High level overview of 5G and Industry 4.0
[20]	1	1		reliability	real-world demonstration	5G in a real-world industrial application
[21]	1	2	2	reliability	system architecture	5G communication system architecture for manufacturing
[22]		1		reliability	real-world demonstration, measurements	URLLC for a distributed control system of a robot
[29]	2	3	2	MEC	architecture	5G for IIoT in smart manufacturing
[30]	1	1		– none –	– none –	IoT in Construction Management
[31]				mmWave, MIMO, beamforming	– none –	Positioning using 5G
[34]		1		reliability	system architecture	5G system architecture for tactile internet
[35]		1		Edge-cloud, network slice	architecture	5G for telesurgery
[36]				NFV	architecture, emulated prototype	NFV in smart manufacturing prototype
[37]				NFV	real-world demonstration, prototyping framework	NFV in smart manufacturing demonstration
[38]				network slicing	system architecture	5G network slicing framework for Industry 4.0
[39]				NFV, network slicing	framework, simulation	Framework and simulation for 5G autonomous vehicles
[40]				MEC	real-world demonstration	Control of a robot arm in MEC

**Table 3:** 5G Use-Case Requirements in Edge Computing.

	Delay	Bandwidth	User Mobility	Energy Consumption	Research Efforts
Tactile internet	<=5 ms	∼1 Gbps	✓	✓	[32,33]
Data/video analytics	∼10 ms	100 kbps–10 Mbps	✓	-	[49,50,51]
XR and Industry 4.0	∼20 ms	∼50 Mbps	-	✓	[52,54,55,56]
Caching in the edge	-	20 Mbps	-	✓	[57,58]
Smart vehicles	<=100 ms	∼20 Mbps	✓	✓	[59,60,61,62]

**Table 4:** Comparison of network solutions.

	Kernel socket		RDMA		DPDK	
Event mode latency (128 / 8192 bytes) (μs)	∼20	∼53	∼15	∼23	∼17	∼28
Poll mode latency (128 / 8192 bytes) (μs)	∼16	∼37	∼5	∼15	∼5	∼15
Hardware capabilities	Commodity		RDMA capable		Supported but not by all	
GPU virtualization compatibility	Yes		Yes			
Physical network capability	Yes		Yes		Yes	
Virtualized underlay network capability	Yes		Yes		Yes	

**Table 5:** Capabilites of Smart NICs and FPGAs.

	SmartNIC	FPGA
Stateless task offloading	Yes	Yes
Offloading general tasks	Yes	No

**Table 6:** Comparison of virtual technologies.

	Virtual Machines	Containers	Unikernels
Startup time	High	Low	Low
Size	Large	Small	Small
Secure	Yes	No	Yes
Used for FaaS	No	Yes	Yes

**Table 7:** Security threats in automation IoT and their possible mitigation [83].

Layer	Threat type	Mitigation
Physical	Tampering	tamper-resistant packaging
	Denial of Service	spread-spectrum techniques
Networking	Denial of Service	active firewalls, passive monitoring (probing), traffic admission control, bi-directional link authentication
	Eavesdropping	encryption, authorization
Data processing	Back door attack	properly configured firewalls on all system entry point
	Social Engineering	educating employees to security awareness
	Exhaustion	traffic monitoring
	Malware	malware detection
Application	Client app.	anti-virus filtering
	Comm. channel	proper authentication, authorization, integrity verification
	Integrity	testing
	Modifications	validation
	Multi-user access	process planning and design
	Data access	Traceability

**Table 8:** Security challenges in 5G technologies [89].

Security Threats	Target Point/Network Element	Effected Technology				Privacy
		SDN	NFV	Channels	Cloud	
DoS attack	Centralized control elements	✓	✓		✓	
Hijacking attacks	SDN controller, hypervisor	✓	✓			
Signaling storms	5G core network elements			✓	✓	
Resource (slice) theft	Hypervisor, shared cloud resources		✓		✓	
Configuration attacks	SDN (virtual) switches, routers	✓	✓			
Saturation attacks	SDN controller and switches	✓			
Penetration attacks	Virtual resources, clouds		✓		✓	
User identity theft	User information data bases				✓	✓
TCP level attack	SDN controller-switch communication	✓	✓			
Man-in-the-middle attack	SDN controller-communication	✓		✓		✓
Reset and IP spoofing	Control channels			✓		
Scanning attacks	Open air interfaces			✓		✓
Security keys exposure	Unencrypted channels			✓		
Semantic information attacks	Subscriber location			✓		✓
Timing attacks	Subscriber location				✓	✓
Boundary attacks	Subscriber location					✓
IMSI catching attacks	Subscriber identity			✓		✓

**Table 9:** Some advantages and drawbacks of blockchain usage when compared to traditional data handling.

Area to Consider	Advantage	Drawback
Ledgers	Ledgers as distributed and trusted databases	Mixed usage of private and public blockchains
		are not clearly solved in practice
Transaction speed	Accelerated transactions when compared	Number of transactions
	to industrial bulk commissioning	are still less than 100 per second
Transaction as event logging	Possibility of micropayments	Limitations for resource constrained devices
Security	Advanced, inherited security	Security vulnerabilities, such as
	Trust	51% attack
	Data Privacy	race attack, finney attack,
	Confidentiality, Integrity, Availability	bugs in Smart Contracts are not patchable
Leaving out third parties	Anonymity	Legal issues
	Cost reductions by removing middlemen	are not easy to solve

**Table 10:** Deep Learning application examples of FCAPS Management tasks in the mobile telecommunications domain [120].

Management Area	Prediction	Anomaly Detection	Clustering or Classification
**Fault management**	Fault Prediction;	Fault Detection;	Alarm Correlation
	Automated Mitigation	Root Cause Analysis	
**Configuration m.**	Resource Optimization	Configuration	Realizing similarities
	(SDN, Base Station power adj.,	pattern	or differences
	Cloud resource allocation)	recognition	in node configs
**Accounting m.**	Churn prediction;	Misuse or Fraud	Traffic characterization;
	Service utilization prediction		Usage profiling
**Performance m.**	Utilization prediction	Detecting under- or	Resource Planning
	(feeding Config. mgmt.)	over-utilization of segments	QoS and QoE correlation
**Security mgmt.**	Intrusion Prevention	Detection of suspicious activities	Intrusion Detection
		DDoS Detection	

**Table 11:** AI technologies in various layers of 5G systems.

Area	Use Case	Related Work
Service management	operations support systems	[114,115]
	resource provisioning	[116]
	fault localization, failure root cause analysis	[117,120]
	security	[118,119]
Network and cloud resource management	flexible function deployment	[115,121]
	network elasticity	[122]
Radio management	radio channel	[123,124]
	user mobility	[125]
	air interface coordination	[126]
